# Punctuated chromatin states regulate Plasmodium *falciparum* antigenic variation at the intron and 2 kb upstream regions

**DOI:** 10.1186/s12864-016-3005-7

**Published:** 2016-08-18

**Authors:** Chengqi Wang, Swamy R. Adapa, Justin Gibbons, Stephen Sutton, Rays H. Y. Jiang

**Affiliations:** 1 Department of Global Health (GH) & Center for Drug Discovery and Innovation (CDDI), College of Public Health, University of South Florida, Tampa, FL 33612 USA; 2 Department of Molecular Medicine, University of South Florida, Tampa, FL 33612 USA

**Keywords:** *Var* gene, 2 kb upstream region, Intron, FAIRE-Seq, MNase-Seq, Chromatin accessibility

## Abstract

**Background:**

Understanding the regulation mechanism of *var* gene expression is crucial for explaining antigenic variation in *Plasmodium falciparum*. Recent work observed that while all *var* genes produce transcripts, only a few *var* genes exhibit high expression levels. However, the global regulation of *var* expression and the relationship between epigenetic and genetic control remains to be established.

**Result:**

We have systematically reanalyzed the existing genomic data including chromatin configurations and gene expressions; and for the first time used robust statistical methods to show that the intron and 2 kb upstream regions of each endogenous *var* gene always maintain high chromatin accessibility, with high potential to bind transcription factors (TFs). The levels of transcripts for different *var* gene family members are associated with this chromatin accessibility. Any given *var* gene thus shows punctuated chromatin states throughout the asexual life cycle. This is demonstrated by three independent transcript datasets. Chromatin accessibility in the *var* intron and 2 kb upstream regions are also positively correlated with their GC content, suggesting the level of *var* genes silencing might be encoded in their intron sequences. Interestingly, both *var* intron and 2 kb upstream regions exhibit higher chromatin accessibility when the genes have relatively lower transcription levels, suggesting a punctuated repressive function for these regulatory elements.

**Conclusion:**

By integrating and analyzing epigenomic, genomic and transcriptomic data, our work reveals a novel distal element in *var* control. We found dynamic modulations of specific epigenetic marks around the *var* intron and distal upstream regions are involved in the general *var* gene expression patterns in malarial antigenic variation.

**Electronic supplementary material:**

The online version of this article (doi:10.1186/s12864-016-3005-7) contains supplementary material, which is available to authorized users.

## Background

*Plasmodium falciparum* is responsible for 85 % of malaria cases and kills over one million people each year [1]. The most prominent virulent surface antigen in *P. falcuparum* is the protein PfEMP1 (*P. falciparum* erythrocytic membrane protein 1) encoded by the *var* multi-copy gene family [2–5]. To escape host immune detection, only small subsets of var genes are expressed at a time [6, 7]. Therefore, understanding the silencing mechanism of *var* genes is critical in the fight against *P. falciparum*.

Research over the past 10 years has identified *var* intron and promoter elements as key for *var* gene silencing. Daily et al. first observed that a *luciferase* gene can be silenced by placing a *var* intron at the gene’s 3′ end [8]. Using transfected plasmids Voss et al. found that a *var* gene upstream region is capable of silencing a gene thereby promoting mono-allelic expression [9]. Another study showed pairing a *var* promoter with a *var* intron or with a different promoter can repress *luciferase* gene expression suggesting *var* promoter silencing is similar to *var* intron-mediated silencing [10]. A model for intron-mediated silencing was proposed by Frank et al., which showed *var* promoters paired with a *var* intron always inhibit *luciferase* gene expression [11]. This result suggests a model that requires a one-to-one pairing requirement between *var* promoters and *var* introns for gene silencing. Recent studies using transfected plasmids provide more evidence that promoter and intron interaction is essential for *var* gene silencing [12, 13].

Other studies have suggested the heterochromatin marker H3K36me3 is involved in *var* gene silencing. Earlier work using high resolution ChIP-chip analysis demonstrated a link between *var* gene silencing and H3K9me3 levels [14]. Recent work revealed the heterochromatin marker H3K36me3 is present along the entire *var* gene body and *var* gene expression is associated with relatively lower H3K36me3 level [15].

Spatial organization also plays a critical role for *var* gene silencing [16]. Most *var* genes are tethered to the nuclear periphery, even though some of them are located on the chromosomal center region [14, 17]. A repeat region within the *var* intron is crucial for retaining a distinct perinuclear position [18]. Epigenetic research also shows that the heterochromatin marker H3K9me3 is associated with genes localized to the nuclear periphery [14]. Hence, tethering to the nuclear periphery seems to be an intrinsic requirement for *var* gene silencing.

Interestingly all *var* genes can produce transcripts [19, 20], but these transcripts are not polysome-associated [20]. This observation implies a post-transcriptional mechanism for *var* gene silencing. Recent works have proven this hypothesis. Zhang et al. showed an exoribonuclease named as PfRNaseII is involved in nascent *var* RNA degradation [21]. Long non-coding RNA also participates in *var* gene regulation. Antisense long non-coding RNA initiating from the *var* intron is associated with the active *var* gene [21]. Further experimentation demonstrates these antisense transcripts may contribute to remodeling of chromatin conformation along the gene body.

Although the 5′ upstream and intron regions play a central role in the *var* gene silencing mechanism, which epigenetic marker on these two regions is involved in *var* gene silencing remains unknown. *Var* genes have a long 5′ upstream region (longer than 1 kb). However, the location of the *var* regulatory region remains unsolved. Next-generation sequencing (NGS) allows screening of the regulation activity for all DNA elements located in *P. falciparum*. By using FAIRE-Seq (formaldehyde-assisted isolation of regulatory elements–sequencing) [22] and MNase-Seq (MNase-assisted isolation of nucleosomes-sequencing) data [23], we analyzed the chromatin accessibility of each *var* intron. Chromatin accessibility has been widely used for detecting active regulation elements in mammalian genomes [24–26]. RNA-Seq (RNA-sequencing) [20, 27] and cDNA sequencing data [28] were also incorporated to investigate the relationship between regulatory potential of different DNA elements and gene expression levels. Our results provide evidence that *P. falciparum* may have developed a specialized nucleosome landscape as a key mechanism to regulate its *var* gene expression. We have also characterized a specific 5′ upstream region that is associated with *var* gene expression. This work addresses a controversial issue in the field of *var* gene silencing and provides insights into the silencing mechanism of native *var* gene clusters.

## Results

### Genome wide intron scan reveals all *var* gene introns maintain high chromatin accessibility

**Fig. 1:**
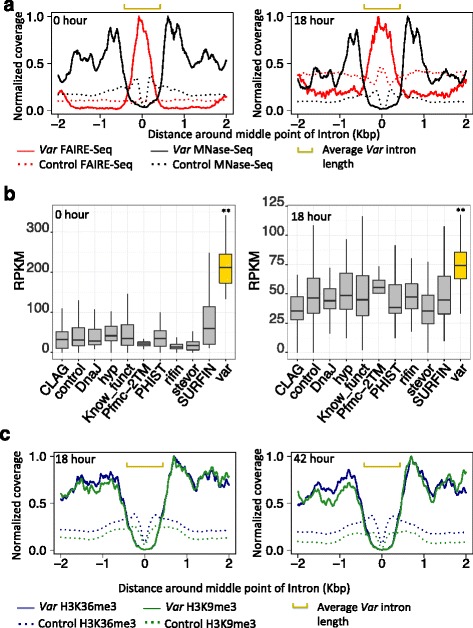
*Var* introns show significantly higher chromatin accessibility. **a** Average genome-wide sequence read coverage around the intron. Both data sets show higher chromatin accessibility around *var* introns. Coverage is expressed as the fraction of the highest coverage value among all positions within the plot window. The yellow bar represents average intron length. **b** Boxplot shows FAIRE-Seq signal around introns grouped according to gene annotation (‘**’ represents *p*-value < 0.01, FDR <0.01 for all comparisons between different gene groups and *var* genes). **c** Average genome-wide heterochromatin sequence read coverage around the intron. Both data sets show lower heterochromatin signal around *var* introns. Coverage is defined as in Fig. 1a

Earlier transgenic works showed that the *var* intron is required for *var* gene silencing [12, 29]. Most researchers reasoned that the regulatory activity of the *var* intron is crucial for *var* gene silencing [10, 16, 30]. However, no study so far has directly investigated the regulatory activity of the endogenous *var* introns. To address this problem, we used FAIRE-Seq [22] at seven time point (hours 0, 6, 12, 18, 24, 30 and 36) and MNase-Seq [23] data at three different time points (hour 0, 18 and 36) throughout the parasite erythrocytic cycle to explore this activity. FAIRE-enriched regions have been shown to correspond to open chromatin states with high regulatory activity [24], On the other hand, MNase-Seq is a powerful method to map the genome wide distribution of nucleosome occupancy. MNase-enriched regions indicate lower regulatory activity [31]. We first observed most *var* genes, no matter which categories (ups A, ups B or ups C), always possess enriched FAIRE-Seq signal in their introns, while all the neighboring regions of introns keep higher enriched MNase-Seq signal during hours 0 and 18 (Additional file 1: Figure S1). However, at hour 36, the neighboring regions of *var* introns also show enriched FAIRE-Seq signal. Figure 1a shows the average FAIRE and MNase signal coverage around all *var* introns at hours 0 and 18 (Additional file 2: Figure S2 shows sequencing signals for hours 6, 12, 24 and 30 and 36, MNase-seq data is only available for hours of 0, 18 and 36). We observed that *var* introns keep significantly higher chromatin accessibility for transcription factor (TF) binding compared with its neighboring regions in ring and trophozoite stages (Wilcoxon-Rank-Sum test *p*-value < 2.2e-16 in hours 0, 6, 12, 18, 24 and 30 compared with all other genes with one intron). In addition, the *var* introns always keep significantly lower nucleosome occupancy in ring, trophozoite and schizont stages (Wilcoxon-Rank-Sum test *p*-value < 2.2e-16 for 0, 18 and 36 compared with all other genes with one intron represented as dash line in Fig. 1a).

We also investigated whether the high regulatory potential of the intron is specific to *var* genes. The chromatin accessibility for different gene families was compared. These families comprise nearly all members of variant surface antigens, *rifin*, *Pfmc*-*2TM* and *stevor,* and other subtelomeric gene families such as *CLAG, SURFIN, and many members of exported protein families such as DnaJ, hyp,* and *PHIST genes.* For *var* genes, we observe a higher enrichment of FAIRE-seq signal (Fig. 1b, Additional file 3: Figure S3 and Additional file 4: Table S1, *p*-value < 0.05, FDR <0.05 during all time points). The significantly higher chromatin accessibility in *var* introns indicates *var* introns exhibit specific regulatory functions. To rule out that this signal enrichment is observed by chance, we assembled all the genes containing a single intron as the control genes. The average sequencing signal around introns of control genes was calculated and plotted as the red dashed line in Fig. 1a and Additional file 2: Figure S2. Compared with the control genes, the *var* introns always show significantly higher FAIRE-Seq, indicating *var* genes always keep high regulatory potential and low nucleosome occupancy.

Heterochromatin markers along the *var* gene body also play a key role in regulation [15]. We checked if the *var* intron keeps high levels of heterochromatin marker similar to the whole gene body. By using recent H3K36me3 and H3K9me3 ChIP-Seq data [15] (Data are only available on hours 18 and 42), we showed the *var* intron maintains low levels of H3K36me3 and H3K9me3 (Fig. 1c, Wilcoxon-Rank-Sum test *p*-value < 2.2e-16, compared with neighboring region). The level of heterochromatin makers around *var* introns is consistent with their open chromatin state, suggesting the *var* intron is involved in the *var* gene regulation mechanism.

### 2 kb upstream region of *var* genes keep high chromatin accessibility

**Fig. 2:**
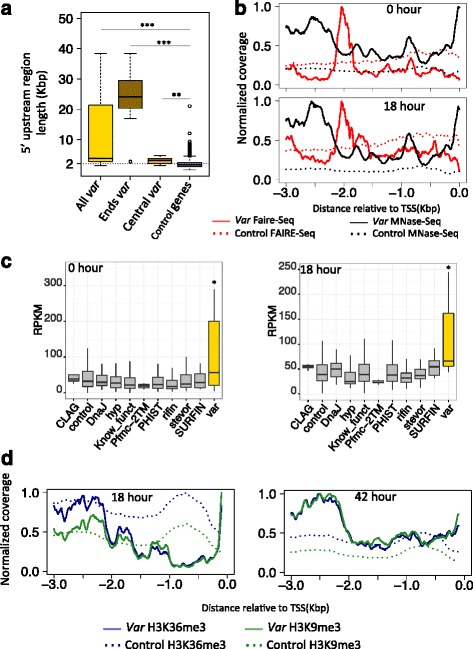
*Var* 2 kb upstream regions show higher chromatin accessibility. **a** Boxplot distribution shows *var* genes have longer 5′ upstream region (Control genes indicate all *P. falciparum* genes except *var* genes). Ends *var* indicates the 25 *var* genes located on the end of chromosome, while central *var* indicates the 37 of *var* genes located on the central region of chromosome (‘**’ represents *p*-value < 0.01, while ‘***’ represents *p*-value < 2.2e-16. P-value was calculated based on Wilcoxon-Rank-Sum test). **b** Average genome-wide sequence read coverage on 5′ upstream region. Both data sets show higher chromatin accessibility around 2 kb upstream region. **c** Boxplot shows FAIRE-Seq signal around the 2 kb upstream regions grouped according to gene annotation (‘*’ represents *p*-value < 0.05, FDR <0.05 for all comparisons between different gene groups and *var* genes). **d** Average genome-wide heterochromatin sequence read coverage on 5′ upstream region. Both data sets show lower heterochromatin signal around 2 kb *var* upsteam regions. Coverage is expressed as Fig. 1a

Earlier works have shown that the *var* 5′ upstream flanking region is another important regulatory element for *var* gene regulation [9, 29, 30]. However, *var* genes have long (>1 kb) 5′ upstream regions compared with other genes (Fig. 2a, Wilcoxon-Rank-Sum test *p*-value < 2.2e-16). Since *P. falciparum* exhibits long rang interactions [32], the regulatory site of *var* genes might be far from the transcription start site (TSS). We used FAIRE-Seq [22] and MNase-Seq [23] data to investigate possible locations of 5′ upstream regulatory elements. We found most *var* genes exhibit high chromatin accessibility around the 2 kb upstream region in ring, trophozoite and schizont stages (Fig. 2b for hours of 0 and 18, Wilcoxon-Rank-Sum test *p*-value < 0.05 compared with all other genes with one intron). Additional file 5: Figure S4 shows FAIRE-Seq for hours 6, 12, 24, 30 and 36. Specific examples are shown in Additional file 6: Figure S5. Except for the 2 kb region, most of the 5′ upstream region of *var* genes shows lower chromatin accessibility and higher nucleosome occupancy during the ring and trophozoite stages (Additional file 7: Figure S6, Wilcoxon-Rank-Sum test *p*-value < 0.05 compared with all other genes with one intron). To test whether the regulatory ability of the 2 kb upstream region is unique to *var* gene regulation, we also compared FAIRE-Seq signal with other gene families (Fig. 2c for hours 0 and 18, and Additional file 8: Figure S7 for hours 6, 12, 24, 30 and 36). The results show the 2 kb upstream region of *var* genes exhibit significantly higher chromatin accessibility during ring and trophozoite stages (Wilcoxon-Rank-Sum test *p*-value < 0.05, FDR <0.05 in hours 0, 6, 12, 18, 24 and 30, Additional file 9: Table S2). The heterochromatin ChIP-Seq signal around the 5′ upstream region also supports our conclusion for hour 18 (Fig. 2d), with *var* genes showing a clear valley at the 2 kb upstream region. All these results suggest that the *var* 2 kb upstream regions are potential regulation regions and may be involved in the *var* gene regulation mechanism.

### The chromatin accessibility around the 2 kb upstream region and intron is associated with *var* gene expression level

**Fig. 3:**
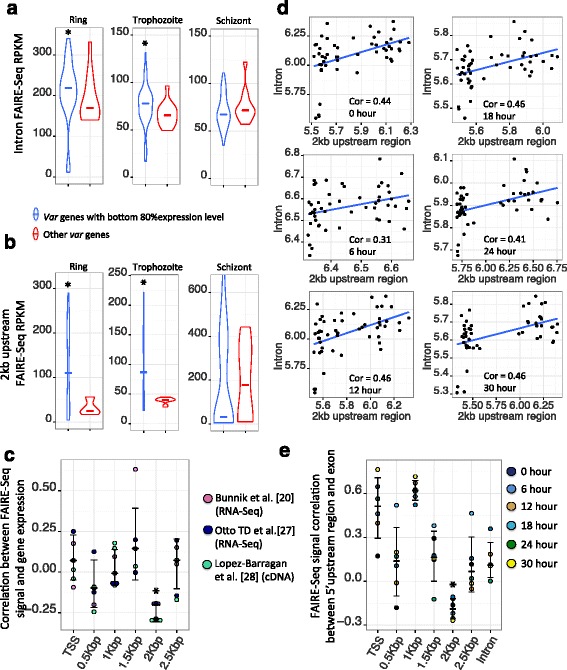
The chromatin accessibility of introns and 2 kb upstream region are associated with var gene expression. **a**, **b** Vioplots show the FAIRE-Seq signals of different *var* gene regions that are classified based on gene expression data (Otto TD et al. RNA-Seq data [27]). **c** The bee swarm plot shows Pearson correlation value between FAIRE-Seq signal on different *var* 5′ upstream regions and *var* gene expression from three independent expression data sets. The median value and standard deviation is represented by the black bar (‘*’ represents Wilcoxon-Rank-Sum test *p*-value < 0.05 compared with the second most significant correlation value). **d** Scatterplot shows FAIRE-Seq signal around *var* intron and 2 kb upstream region. X-axis and Y-axis represent *log*(FAIRE-Seq RPKM) value on *var* 2 kb upstream and intron region. **e** The bee swarm plot shows FAIRE-Seq signal correlation value between different *var* regions and exon during ring and trophozoite stages (‘*’ represents Wilcoxon-Rank-Sum test *p*-value < 0.05 compared with second significant correlation value)

Previous works showed *var* gene transcripts could be detected in blood-stage *P. falciparum* [19, 20], though these transcripts were not polysome-associated [20]. In line with these reports [19, 20], we observed most *var* genes produced transcripts using three published expression data sets [20, 27, 28]. We compared the expression of each *var* gene with the expression distribution of all other genes. More than 90 % of *var* genes have a *p*-value bigger than 0.05 indicating *var* gene expression levels are not significantly different than other genes. However, most *var* genes have low expression levels with only one or two of them exhibiting high expression levels (Additional file 10: Figure S8). In addition, the expression pattern between these three data sets is similar (Pearson expression correlation between three dataset >0.33). Therefore, we expected that the *var* genes with low expression levels would be consistent between the independent datasets. To investigate this assumption, *var* genes with the bottom 80 % expression levels were extracted independently from three data sets [20, 27, 28] and most of them consistently appeared in the bottom 80 % expression group (~78 % *var* genes were in the lowest 80 % expression group in all 3 datasets, Fisher’s exact test *p*-value = 2.58e-2 compared with expectation). We investigated whether the bottom 80 % of expressed *var* genes tend to exhibit higher chromatin accessibility around their intron and 2 kb upstream regions. *Var* genes with the bottom 80 % expression level were extracted and their chromatin accessibility around the two possible regulatory regions was compared with the remaining *var* genes. Figure 3a, b shows *var* genes with the bottom 80 % expression level (Otto TD et al. RNA-Seq data [27]) have significantly higher chromatin accessibility compared with the remaining *var* genes in ring and trophozoite stages. Additional file 11: Figure S9 and Additional file 12: Figure S10 shows similar result using Bunnik et al. [20] and Lopez-Barragan et al. [28] expression data. In addition, we also investigate whether other 5′ upstream regions including TSS, 0.5 kb, 1 kb, 1.5 kb and 2.5 kb upstream regions of *var* genes are also involved in regulation (Data are available in Additional file 13: Table S3 and Additional file 14: Table S4). The Pearson correlation values between FAIRE-Seq signals around these upstream regions and *var* gene expression was calculated. *Var* genes having higher expression levels show lower chromatin accessibility on two regulatory regions (2 kb upstream region and intron) during ring and trophozoite stages, seven correlation values for these two blood stages were calculated from three different expression data sets (2 for Bunnik et al. data [20], 2 for Otto TD et al. data [27] and 3 for Lopez-Barragan et al. data [28]). As shown in Fig. 3c, the chromatin accessibility only in the 2 kb upstream region shows negative correlation with *var* gene expression (Wilcoxon-Rank-Sum test *p*-value < 0.05 compared with all other upstream regions), suggesting only the 2 kb upstream region of *var* genes is associated with *var* expression level.

The “promoter-intron pairing model” suggests *var* gene regulation requires the presence of both the *var* promoter and intron [10, 11]. To test this model, we checked the correlation of chromatin accessibility between the *var* 5′ upstream region and the intron. As shown in Fig. 3d, the *var* 2 kb upstream region and the intron have significant positive Pearson correlation values during ring and trophozoite stages (Pearson correlation >0.31 for all time points, Bootstrap and Wilcoxon-Rank-Sum test *p*-value < 2.2e-16 compared with other genes with only one intron). Other 5′ upstream regions are also considered and the correlation value with the *var* intron calculated (Additional file 15: Figure S11). The result shows only the 2 kb upstream region exhibits significant chromatin accessibility correlation with the *var* intron (Wilcoxon-Rank-Sum test *p*-value < 0.05 compared with all other upstream regions), suggesting a “punctuated chromatin” status on intron and 2 kb upstream regions.

“Chromatin spreading” is another model for explaining *var* gene regulation, which states lower expressed *var* genes require the presence of heterochromatin, such as H3K36me3 along the entire gene body [15]. “Pairing” and “chromatin-spreading” models are not necessarily mutually exclusive. Here, we investigated whether the chromatin accessibility around *var* regulatory regions (2 kb upstream and intron) is associated with the heterochromatin marker along *var* exons. As shown in Fig. 3e, only the 2 kb upstream region of *var* genes shows significant negative chromatin accessibility correlation with *var* exons during ring and trophozoite stages (Wilcoxon-Rank-Sum test *p*-value < 0.05 compared with all other *var* regions), implying the regulatory ability around 2 kb upstream region may be associated with the chromatin accessibility around the *var* exon region. This result is also supported by H3K36me3 ChIP-Seq data [15] from the trophozoite stage, the FAIRE-seq signal of 2 kb upstream regions is positively correlated with heterochromatin H3K36me3 level around the *var* exons (Pearson correlation = 0.25, Bootstrap and Wilcoxon-Rank-Sum test *p*-value < 0.05 compared with other *var* regions). Interestingly, the chromatin accessibility around the 1 kb upstream region of *var* genes exhibits positive correlation with its exon during stages of ring and trophozoite (Fig. 3e), though it is not significant compared with TSS region (Wilcoxon-Rank-Sum test *p*-value = 0.15). This region was previously reported to have the H2A.Z marker enriched in this region of active *var* genes [33]. However, the 1 kb upstream region does not show significantly higher chromatin accessibility compared with its neighboring region (Wilcoxon-Rank-Sum test *p*-value > 0.35). Higher resolution chromatin accessibility data is required to test whether the chromatin accessibility around this region is associated with the chromatin marker of the *var* gene exon region.

### The GC content of *var* regulation regions is associated with their chromatin accessibility

**Fig. 4:**
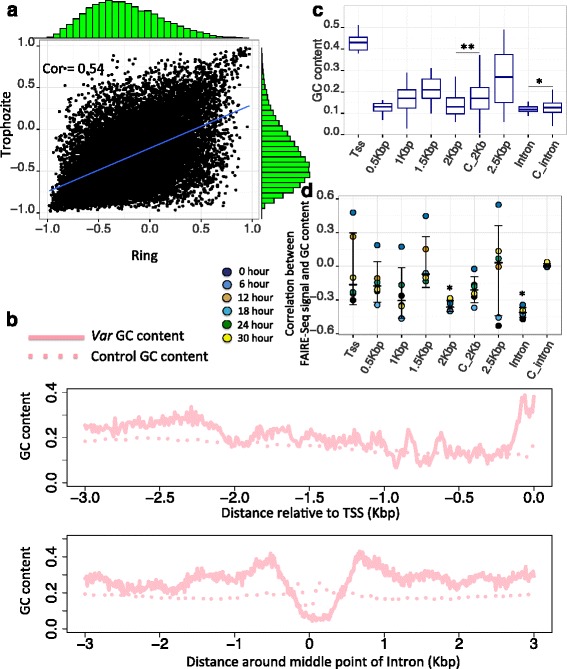
Sequence GC content on two *var* regulatory regions is associated with their chromatin accessibility. **a** Scatterplot shows the correlation between sequence GC content and chromatin accessibility of genome 1 kb bins during ring and trophozoite stages (see Methods). Each point represents a 1 kb bin in P. falciparum genome. X-axis and Y-axis represent the correlation value between GC content of each 1 kb bin (each bin is divided again into 10 bins with length 100 bp) and its FAIRE-Seq signal during ring and trophozoite stages. Green histogram plot represents the distribution of correlation value. **b** Average sequence GC content around *var* intron and 2 kb upstream regions is plotted. **c** The sequence GC content distribution of different genome regions are plotted (‘C_2kb’ and ‘C_intron’ indicate 2 kb upstream and intron region of P. falciparum genes with one intron, ‘*’ represents Wilcoxon-Rank-Sum test *p*-value < 0.05). **d** The bee swarm plot shows Pearson correlation between sequence GC content of different genome regions and their FAIRE-Seq signal during different blood stages (‘*’ represents Wilcoxon-Rank-Sum test *p*-value < 0.05 compared with other elements)

In an earlier study, Bunnik and coworkers reported AT-repeats segments strongly inhibit nucleosome occupancy (nucleosome occupancy is measured by MNase-seq) [23], implying that a correlation between GC content and their potential for chromatin accessibility. To see if the chromatin accessibility of the *P*. f*alciparum* genome is uniformly correlated with GC content the correlation between GC content and chromatin accessibility during ring and trophozoite stages was calculated (see Methods, Fig. 4a). The FAIRE-Seq signal along the majority of *P*. f*alciparum* genome region is anti correlated with its GC content (79 and 83 % genome region during ring and trophozoite stage have a correlation value lower than −0.1). The correlation value between chromatin accessibility and GC content is conserved during different blood stages (Pearson correlation value = 0.54, Wilcoxon-Rank-Sum test *p*-value < 2.2e-16 compared with the correlation value of shuffled genome, see Methods). However, not all genome regions show a negative correlation between chromatin accessibility and GC content (Fig. 4a). It is necessary to check whether the functional region of *var* genes also exhibit this anti-correlation between GC content and an open chromatin state. We first plotted the landscape of GC content around two *var* regulatory regions (Fig. 4b, c). The results show *var* 500 bp upstream, 2 kb upstream and intron regions show relatively lower GC content (Wilcoxon-Rank-Sum test *p*-value < 2.2e-16 compared with their neighboring region, Wilcoxon-Rank-Sum test *p*-value < 0.05 compared with control genes), implying the higher chromatin accessibility along *var* regulatory regions might be encoded in their sequence information. To further support this conclusion, the Pearson correlation between chromatin accessibility along two *var* regulatory regions during ring and trophozoite stages and their sequence GC content was calculated. There is a significant anti-correlation value on the *var* 2 kb upstream and intron regions (Fig. 4d, Wilcoxon-Rank-Sum test *p*-value < 0.05 compared with all other regions), suggesting the sequence GC content is associated with the chromatin accessibility around *var* regulatory regions.

To exclude the possibility that the higher chromatin accessibility in the *var* intron and 2 kb upstream regions maybe due to the intrinsic lower GC content, we extracted intron and 2 kb upstream regions from *P. falciparum* genes based on their GC content distribution in the *var* intron and 2 kb upstream regions. This procedure was repeated 100 times and Additional file 16: Figure S12a and Additional file 17: Figure S13a show the same distribution of GC content on intron and 2 kb upstream regions between control group and *var* genes. We observed *var* intron and 2 kb upstream regions exhibit significantly higher FAIRE-Seq signal compared with control groups during all stages (Additional file 16: Figure S12b and Additional file 17: Figure S13b, *p*-value < 0.01 for all stages).

## Discussion

The silencing of *var* genes is regulated via multiple mechanisms: (i) DNA regulatory elements such as *var* promoters and *var* introns [9, 34], (ii) histone modifications and epigenetic memory [15, 35, 36], (iii) change in subnuclear localization [14, 17], and (iv) post-transcriptional non-coding RNA [21, 37]. These different regulation mechanisms are not necessarily mutually exclusive, and all of them may be at work. It has been demonstrated that the *var* intron is involved in mechanism (iii) [18] and (iv) [37]. However, which epigenetic marker along the intron is involved in *var* gene silencing remains elusive. Similarly, although the *var* 5′ upstream region is an essential element for *var* gene silencing, it is difficult to identify an exact regulatory site in such a large region (>1 kb). Using next-generation sequencing (NGS) we can directly monitor how the epigenetic environment changes, in particular during the different stages of *P. falciparum*’s erythrocytic cycle. We can use NGS data to study the general properties of *var* gene regulation. By using FAIRE-Seq [22] and MNase-Seq [23], we found both *var* intron and 2 kb upstream regions show high chromatin accessibility during ring and trophozoite stages, suggesting high regulatory activity on these two regions. Further investigation shows the chromatin accessibility during these two stages is anti-correlated with *var* gene expression levels with the schizont stage being the only exception. One possible explanation for this exception is that an uncharacterized transcriptional repressor stage specifically binds these potential regulatory regions during ring and trophozoite stages, but not during the schizont stage. A future study using ChIP-Seq (Chromatin Immunoprecipitation Sequencing) could prove this hypothesis. Here, we provide DNA sequence evidence for such a regulator. The MEME algorithm [38] was used to search sequence motifs on *var* intron and 2 kb upstream regions. Two GC-rich motifs were found on the *var* introns (e-value = 1.1e-192) and two GC-rich motifs were also found on the 2 kb upstream regions (e-value = 8.1e-282). Additional file 18: Table S5, Additional file 19: Table S6, Additional file 20: Figure S14 and Additional file 21: Figure S15 list the most statistically significant locations (*p*-value < 1e-8) for these motifs.

**Fig. 5:**
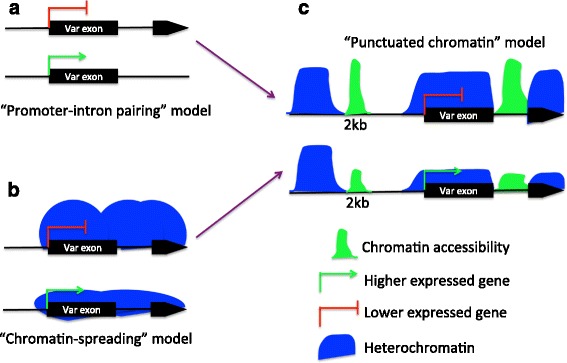
Summary of *var* gene silencing model. **a** “Promoter-intron pairing” model. The *var* intron and promoter are required for *var* gene silencing. **b** “Chromatin-spreading” model. Lower expressed *var* gene exhibits higher heterochromatin marker levels along the gene body. **c** “Punctuated chromatin” model. Both “Chromatin-spreading” and “Promoter-intron pairing” work for *var* gene regulation. Lower expressed *var* genes tend to exhibit higher chromatin accessibility on both intron and 2 kb upstream regions, whereas lower chromatin accessibility can be observed on higher expressed *var* genes

Interestingly, *var* genes tend to exhibit open chromatin on both the *var* intron and 2 kb upstream regions. This result partly supports the “promoter-intron pairing” model were the promoter and intron need to be in an open chromatin state to repress *var* gene expression (Fig. 5a). Except for the *var* intron and 2 kb upstream regions, most *var* regions including the exons and TSS regions contain heterochromatin marker and exhibit lower chromatin accessibility (Fig. 5b). Both the “promoter-intron pairing” and “chromatin spreading” model explain aspects of *var* gene regulation. Since the *var* intron and 2 kb upstream regions tend to show punctuated chromatin states, we propose a new regulation mechanism called the “punctuated chromatin” model (Fig. 5c) that combines the “promoter-intron pairing” and “chromatin spreading” models. Although we observed epigenetic marker association between the 2 kb upstream region and this *var* exon this result still requires more evidence for validation. The “punctuated chromatin” model is consistent with the model proposed by, Avraham et al., which is based on the “promoter-intron pairing” model, were a chromatin loop between the *var* intron and promoter regions plays a role in maintenance of *var* gene silencing [13]. Simultaneous open chromatin states on both the intron and 2 kb upstream regions is in line with this loop model, which creates an ideal epigenetic environment for transcription factor binding. But, directly proving the existence of looping requires higher resolution chromosome conformation capture (3C) data.

In this work, we show that the GC content throughout the genome is not associated with nucleosome occupancy everywhere. In *var* genes, only the GC content of the introns and 2 kb upstream regions is anti correlated with their chromatin accessibility. This result suggests the regulatory ability on these two regions is encoded in their sequences.

## Conclusions

In conclusion, the chromatin accessibility of all *var* regions was analyzed systematically in our work to study the general properties of *var* gene regulation as a group. By using FAIRE-Seq and MNase-Seq data, we first showed that relatively lower chromatin accessibility in both the intron and 2 kb upstream regions is associated with higher *var* gene expression. The chromatin accessibility in these two *var* regulatory regions is positively correlated with their GC content, suggesting the level of *var* gene silencing may be encoded in their sequences. Interestingly, the chromatin accessibility of *var* introns and promoters shows a positive correlation, indicating some transcription factors might bind both. This result agrees with the previous hypothesis that a chromatin loop between the *var* intron and promoter might maintain gene silencing. We found that chromatin punctuation at a novel upstream element and the intron contributes to the *var* gene silencing mechanism promoting malaria antigenic variation.

## Methods

### Dataset and software package

In this work, two complementary high-throughput sequencing datasets, one created via formaldehyde-assisted isolation of regulatory elements sequencing (FAIRE-Seq) [22] and the other created using MNase-mediated purification of mononucleosomes sequencing(MNase-Seq) [23], were used to detect the chromatin accessibility of different genome regions. The data sets can be downloaded from the Short Read Archive (SRA010122 and SRA091359; http://www.ncbi.nlm.nih.gov/sra). H3K36me3 and H3K9me3 Chromatin immunoprecipitation followed by high-throughput DNA sequencing (ChIP-Seq) data [15] were used to detect heterochromatin markers. All the sequencing reads were mapped to *P. falciparum* 3D7 genome v 9.0 using BWA [39]. Using MNase-Seq procedure [40] and approximate nucleosome length [41], all the MNase-Seq sequencing reads are extended from the 5′ end for 150 bp (~1nucleosome length) toward the 3′ direction. The middle 75 nucleotides were extracted to represent the enrichment of the nucleosome signal. This extending procedure was also implemented in recent work [42]. Three independent gene expression data sets [20, 27, 28] normalized using the method described in Ferhat et al. [32] was used in our work to investigate gene expression level during *P. falciparum*’s erythrocytic cycle. The flanking regions [−200 bp, +200 bp] centered at different 5′ upstream sites TSS, 500 bp, 1 kb, 1.5 kb, 2 kb and 2.5 kb were extracted for chromatin accessibility investigation.

For sequence motif discovery, the MEME algorithm [38] was used to search for the most significant motifs in the *var* intron and 2 kb upstream regions.

### Sequencing signal analysis

The reads per kilo-base per million reads (RPKM) is calculated for comparing the sequencing signal between different genome elements.

1 $$ RPKM=\left(10\hat{\mkern6mu} 9\times R\right)/\left({R}_{total}\times L\right) $$

Where *R* is the sequencing reads number in a specific genome segment, *L* represents the length of the segment and *R*_total_ is the total mapped reads in the experiment.

The normalized coverage (*NC*) is calculated for plotting the sequencing signal around the *var* intron and 5′ upstream regions. It is expressed as the fraction of the highest coverage value among all bins within the plot window.

2 $$ N{C}_t=\frac{E_t\left({R}_i\right)}{\mathbf{ma}{\mathbf{x}}_{i=1n,t=1,2}\left({E}_t\left({R}_i\right)\right)}i=1,2,3\dots n;\kern1.5em t=1,2 $$

*“t”* is the gene type of sequencing signal, which *t = 1* for *var* genes and *t = 2* for control genes. “*i” indicates the bin id and* “*n”* indicates the total number of investigated bins. *R*_*i*_ is the reads number in a specific bin i, *E*_*t*_*(R*_*i*_*)* is the average number of reads for a specific gene type *t* in bin *i*. _*t* = 1,2_**max**_*i* = 1 : *n*_(*E*_*t*_(*R*_*i*_)) is the maximum average reads number of all gene type *t = 1,2* on all bins *i = 1:n*.

### Whole genome scale correlation between sequence GC content and chromatin accessibility

For the correlation between GC content and chromatin accessibility on the whole genome scale, the *P. falciparum* genome (genome length 23,292,622 bp) is divided into equal length bins of 1 kb. Each 1 kb bin is represented as *B*_*i*_, where *i = 1 to 23,292.* Each *B*_*i*_ is divided again into smaller bins *b*_*i*_ with equal length 100 bp.

3 $$ {B}_i=\left({b}_1,{b}_2,\dots {b}_j\dots {b}_{10}\right) $$

The GC content (*gc*) and FAIRE-Seq normalized reads number (*NRN*) is calculated for each small bin *b*_*i*_. The GC content and FAIRE-Seq signal for each *B*_*i*_ can be represented as two vectors.

4 $$ g{c}_{B_i}=\left(g{c}_{b_1},g{c}_{b_2},\dots g{c}_{b_j}\dots g{c}_{b_{10}}\right) $$

5 $$ NR{N}_{B_i}=\left(NR{N}_{b_1},NR{N}_{b_2},\dots NR{N}_{b_j}\dots NR{N}_{b_{10}}\right) $$

Then the Pearson correlation value for each bin *B*_*i*_ can be calculated between $g{c}_{B_i}$ and $NR{N}_{B_i}$.

### Correlation comparison

To compare the correlation value with control genes, the bootstrap method [43] was implemented to extract random samples of *var* genes and controls with replacement. Then the Wilcoxon signed-rank test is used to compare the median value for the two samples.

### Shuffled genome generation

A Shuffled genome was generated for each chromosome *C*_*i*_*, i = 1…14* with length *L*_*i*_. We assume each nucleotide *n*_*ij*_, *j = 1… L*_*i,*_ independently follows the same distribution. This distribution comes from the percentage of each nucleotide type in *P. falciparum*.

6 $$ \left(\begin{array}{c}\hfill A=0.4\hfill \\ {}\hfill T=0.4\hfill \\ {}\hfill C=0.1\hfill \\ {}\hfill G=0.1\hfill \end{array}\right) $$

## Additional files

Additional file 1: Figure S1. Average genome-wide sequence read coverage around three *var* introns during P.falciparum’s erythroytic cycle. All three types of *var* genes show significant enrichment of FAIRE-seq signals in their introns in hours 0 and 18. (PDF 149 kb) Additional file 2: Figure S2. Average genome-wide sequence read coverage around var introns for hours 6, 12, 24, 30, 36 and 42(MNase-seq data [25] is available for 36 h). (PDF 112 kb) Additional file 3: Figure S3. Boxplot shows FAIRE-Seq signal around introns grouped according to gene annotation (‘**’ represents *p*-value < 0.01 and FDR <0.01 for all compassion between different gene groups and *var* gene; ‘*’ represents *p*-value < 0.05 and FDR <0.05). (PDF 98 kb) Additional file 4: Table S1. Intron FAIRE-Seq signal comparison between *var* genes and different gene families (P-value is calculated based on Wilcoxon-Rank-Sum test, FDR indicates false discovery rate). (XLSX 51 kb) Additional file 5: Figure S4. Average genome-wide sequence read coverage on 5′upstream region for hours 6, 12, 24, 30 and 36 (MNase-seq data [25] is available for hours 0, 18 and 36). (PDF 102 kb) Additional file 6: Figure S5. Boxplot distribution of FAIRE-Seq signal along *var* 5′ upstream region during stages of ring and trophozoite. (‘****’ represents *p*-value < 2.2e-16; ‘**’ represents *p*-value < 0.01; ‘*’ represents *p*-value < 0.05. P-value was calculated based on Wilcoxon-Rank-Sum test between *var* gene and the genes with one intron in P. falciparum). (PDF 144 kb) Additional file 7: Figure S6. Average genome-wide sequence read coverage on gene 5′ upstream region for hours 6, 12, 24 and 30 (MNase-seq data [25] is available for hours 36). (PDF 126 kb) Additional file 8: Figure S7. Boxplot shows FAIRE-Seq signal around 2 kb upstream regions grouped according to gene annotation (‘*’ represents *p*-value < 0.05 and FDR < 0.05 for all comparisons between different gene groups and *var* genes). (PDF 97 kb) Additional file 9: Table S2. 2 kb upstream region FAIRE-Seq signal comparison between *var* genes and different gene families (P-value is calculated based on Wilcoxon-Rank-Sum test, FDR indicates false discovery rate). (XLSX 53 kb) Additional file 10: Figure S8. Bar plots show expression level of *var* genes during ring stage from three independent data sets. Expression level is expressed as the fraction of the highest expression value among all *var* genes. (PDF 55 kb) Additional file 11: Figure S9. Boxplot shows the FAIRE-Seq signals *var* intron regions that are classified based on gene expression data **a**. Bunnik et al. [20] (RNA-Seq). **b**. Lopez-Barragan et al. [28] (cDNA) (PDF 142 kb) Additional file 12: Figure S10. Boxplot shows the FAIRE-Seq signals *var* 2 kb upstream regions that are classified based on gene expression data **a**. Bunnik et al. [20] (RNA-Seq). **b**. Lopez-Barragan et al. [28] (cDNA) (PDF 143 kb) Additional file 13: Table S3. Genome coordinates of different *var* gene upstream regions. (XLSX 56 kb) Additional file 14: Table S4. Genome coordinates of *var* gene regulatory regions and control gene regions (XLSX 136 kb) Additional file 15: Figure S11. The bee swarm plot shows Pearson correlation between FAIRE-Seq signal and different *var* intron and 5′ upstream region signals. (PDF 41 kb) Additional file 16: Figure S12. FAIRE-Seq signal distribution comparison between *var* introns and control introns. Control introns were extracted from the genes in *P. falciparum* with only one intron and based on the GC content distribution of *var* introns. **a**. The GC content distribution of *var* introns and control introns. **b**. Boxplot distribution shows *var* introns exhibit significantly higher FAIRE-Seq signal compared with control introns (‘****’ represents *p*-value < 2.2e-16; P-value was calculated based on Wilcoxon-Rank-Sum test). (PDF 57 kb) Additional file 17: Figure S13. FAIRE-Seq signal distribution on 2 kb upstream region comparison between *var* and control genes. Control genes were extracted from the genes in *P. falciparum* with only one intron and based on the GC content distribution of *var* 2 kb upstream region. **a**. The GC content distribution on 2 kb upstream region of *var* genes and control genes. **b**. Boxplot distribution shows *var* 2 kb upstream regions exhibit significantly higher FAIRE-Seq signal compared with control 2 kb upstream region (‘**’ represents *p*-value < 0.01; ‘***’ represents *p*-value < 1e-10; P-value was calculated based on Wilcoxon-Rank-Sum test). (PDF 58 kb) Additional file 18: Table S5. Significant sequence motif locations in *var* intron regions. (TXT 4 kb) Additional file 19: Table S6. Significant sequence motif locations around *var* 2 kb upstream regions [−200, +200]. (TXT 4 kb) Additional file 20: Figure S14.
**a**. The sequence motif on *var* introns is discovered by MEME algorithm. **b**. Significant sequence motif locations in *var* intron regions. (PDF 277 kb) Additional file 21: Figure S15.
**a**. The sequence motif on *var* 2 kb upstream regions is discovered by MEME algorithm. **b**. Significant sequence motif locations in 2 kb upstream regions [−200, +200]. (PDF 227 kb)

## Abbreviations

ChIP-seq: Chromatin immunoprecipitation followed by high-throughput sequencing
FAIRE-Seq: Formaldehyde-assisted isolation of regulatory elements–sequencing
FDR: False discovery rate
MNase-Seq: MNase-assisted isolation of nucleosomes-sequencing
PfEMP1: *P. falciparum* erythrocytic membrane protein 1
RPKM: Reads per kilo-bases per million reads
