# Supplementary figures and images for: Punctuated chromatin states regulate Plasmodium falciparum antigenic variation at the intron and 2 kb upstream regions
Source: BMC Genomics. 2016 Aug 18;17:652. doi: 10.1186/s12864-016-3005-7 (PMC4990864; doi:10.1186/s12864-016-3005-7)

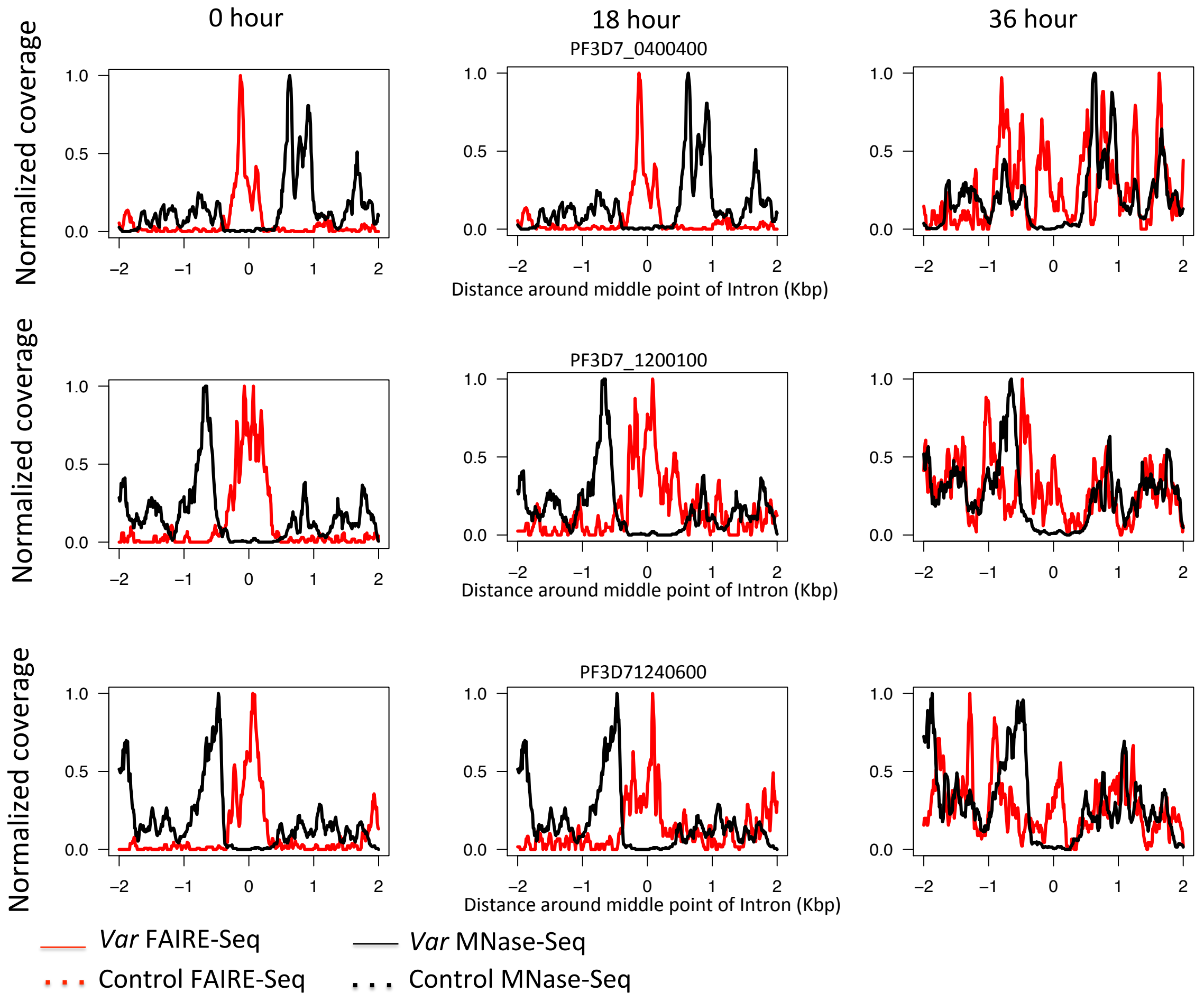

Supplement: Additional file 1: Figure S1. — Average genome-wide sequence read coverage around three var introns during P.falciparum’s erythroytic cycle. All three types of var genes show significant enrichment of FAIRE-seq signals in their introns in hours 0 and 18. (PDF 149 kb) [file 12864_2016_3005_MOESM1_ESM.pdf]

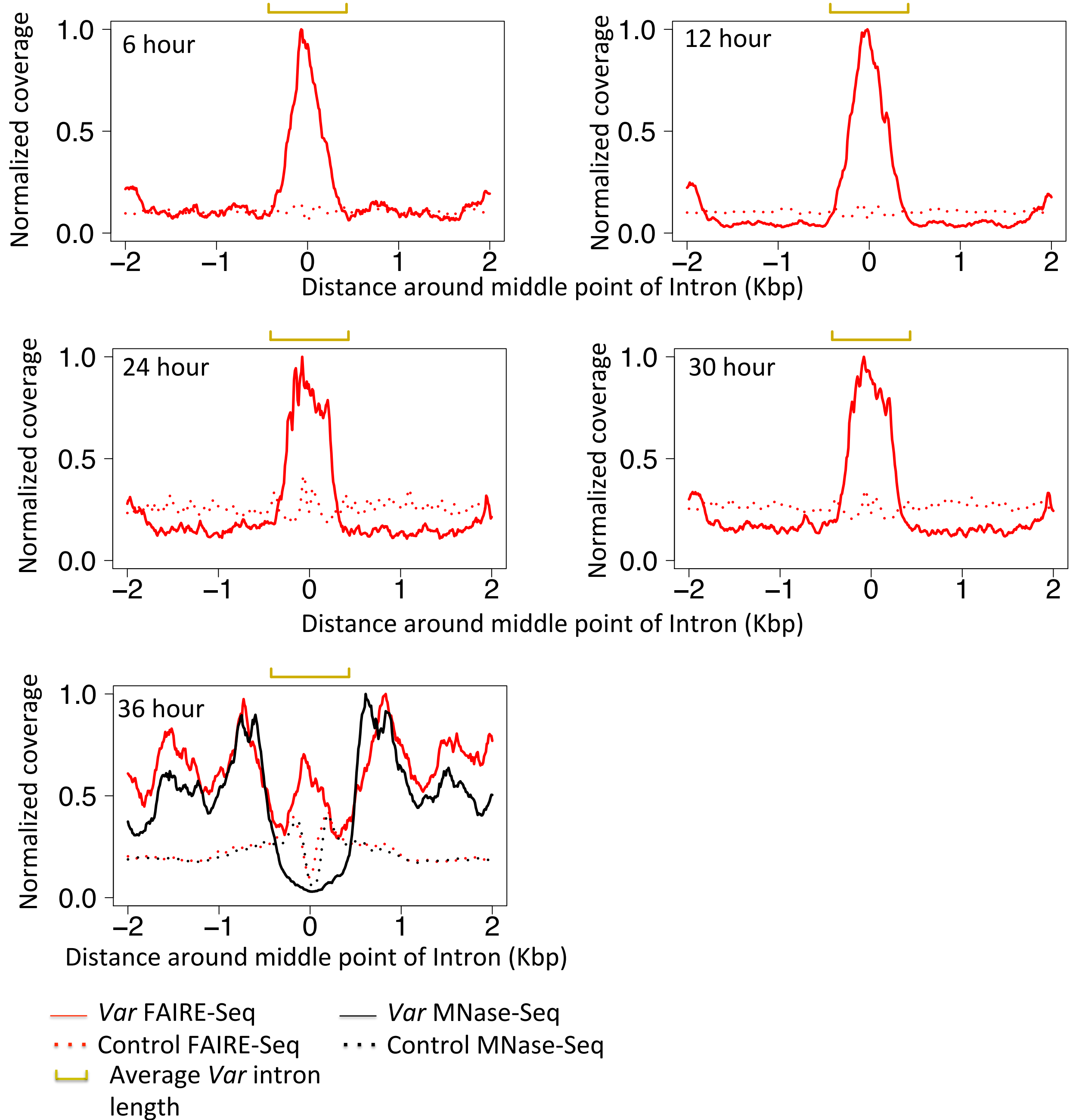

Supplement: Additional file 2: Figure S2. — Average genome-wide sequence read coverage around var introns for hours 6, 12, 24, 30, 36 and 42(MNase-seq data [25] is available for 36 h). (PDF 112 kb) [file 12864_2016_3005_MOESM2_ESM.pdf]

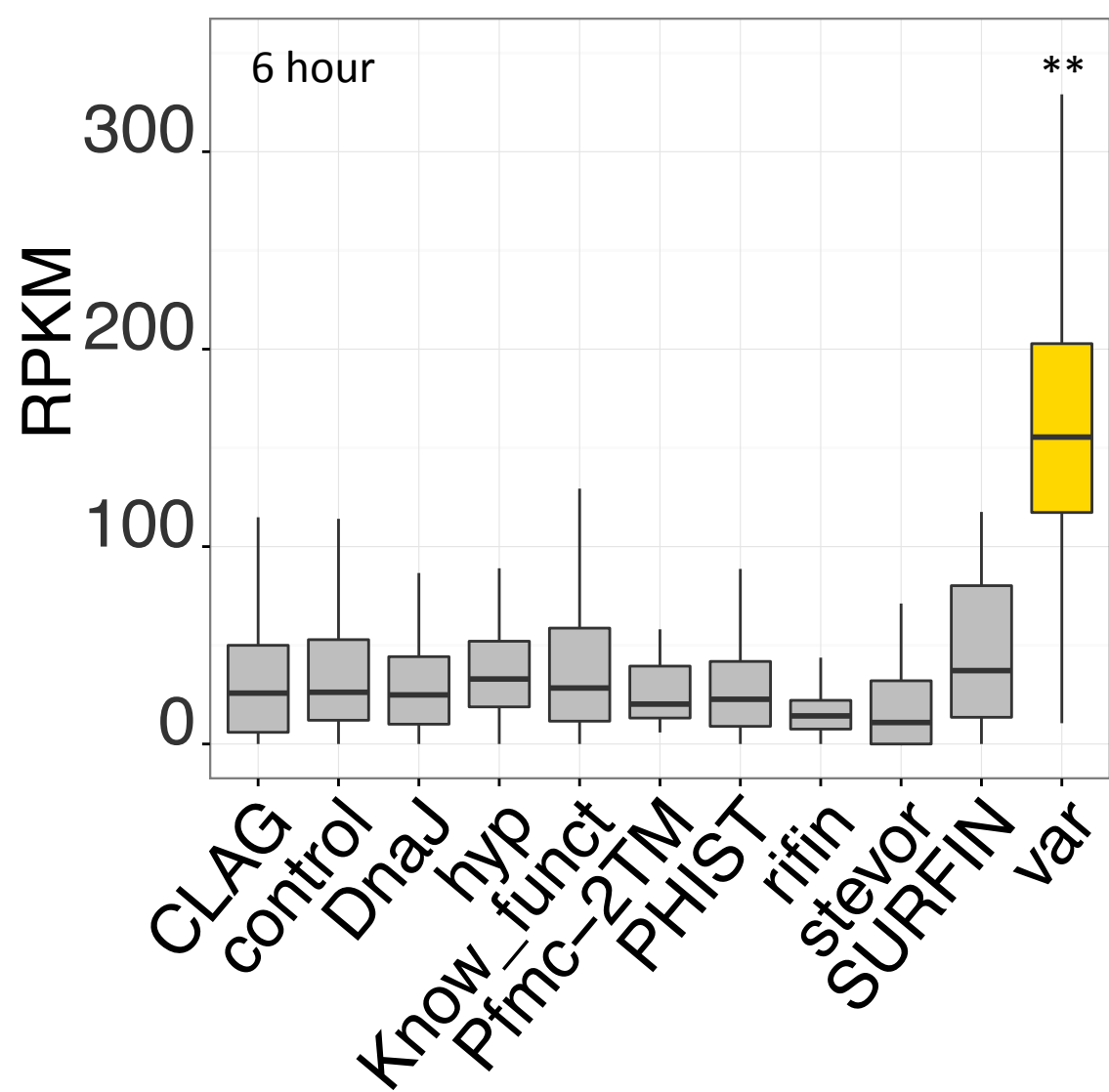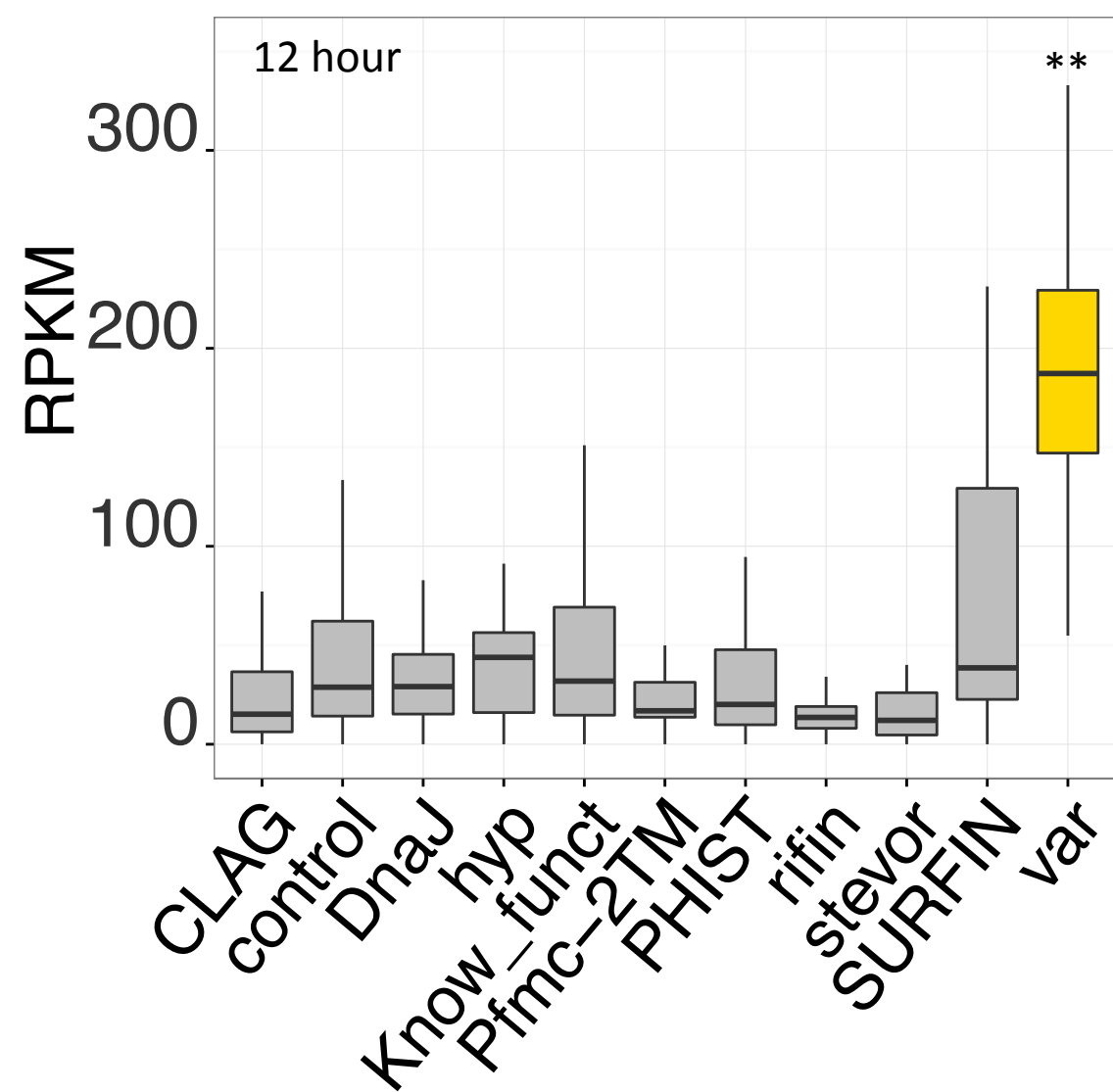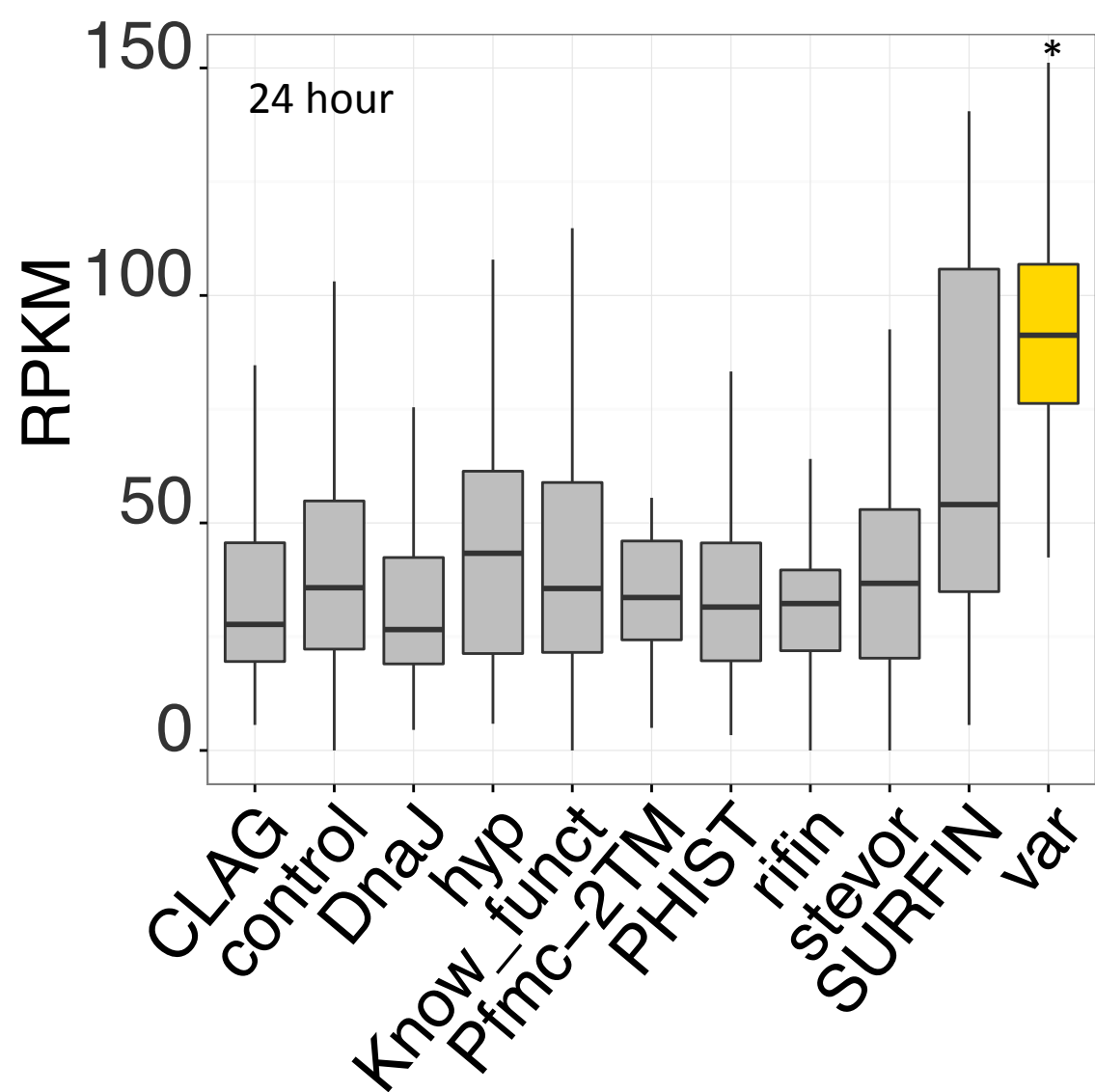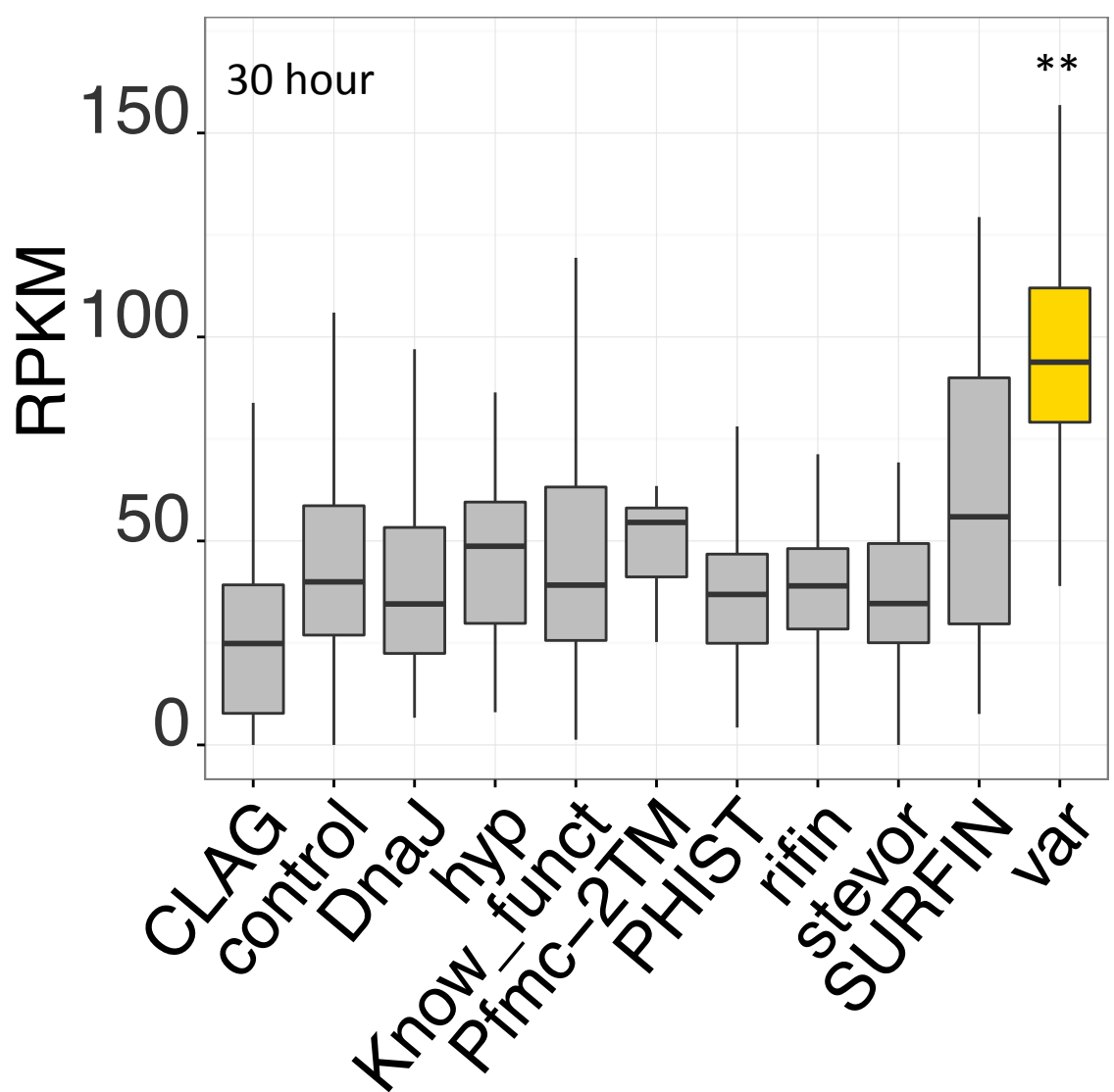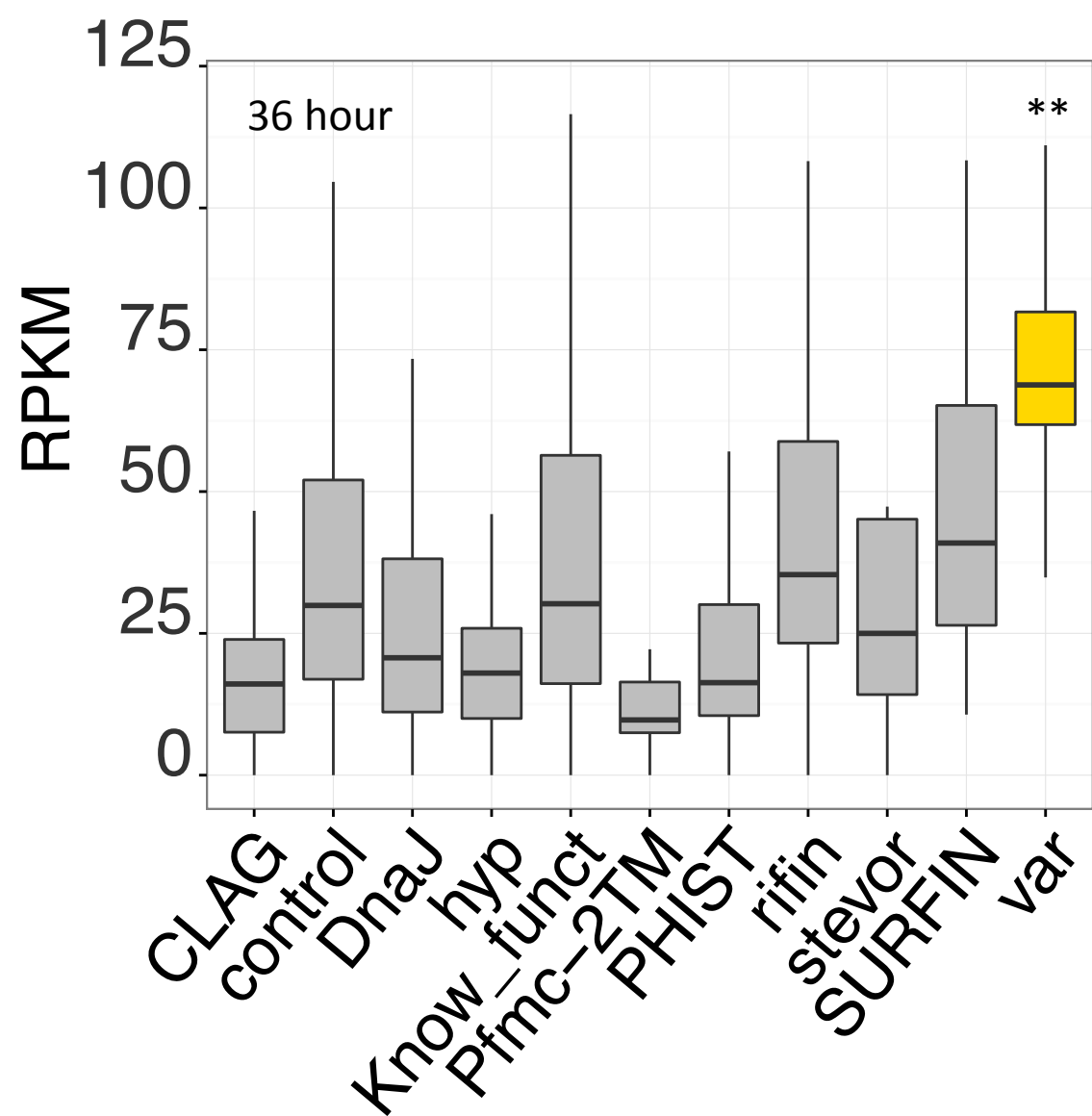

Supplement: Additional file 3: Figure S3. — Boxplot shows FAIRE-Seq signal around introns grouped according to gene annotation (‘**’ represents p-value < 0.01 and FDR <0.01 for all compassion between different gene groups and var gene; ‘*’ represents p-value < 0.05 and FDR <0.05). (PDF 98 kb) [file 12864_2016_3005_MOESM3_ESM.pdf]

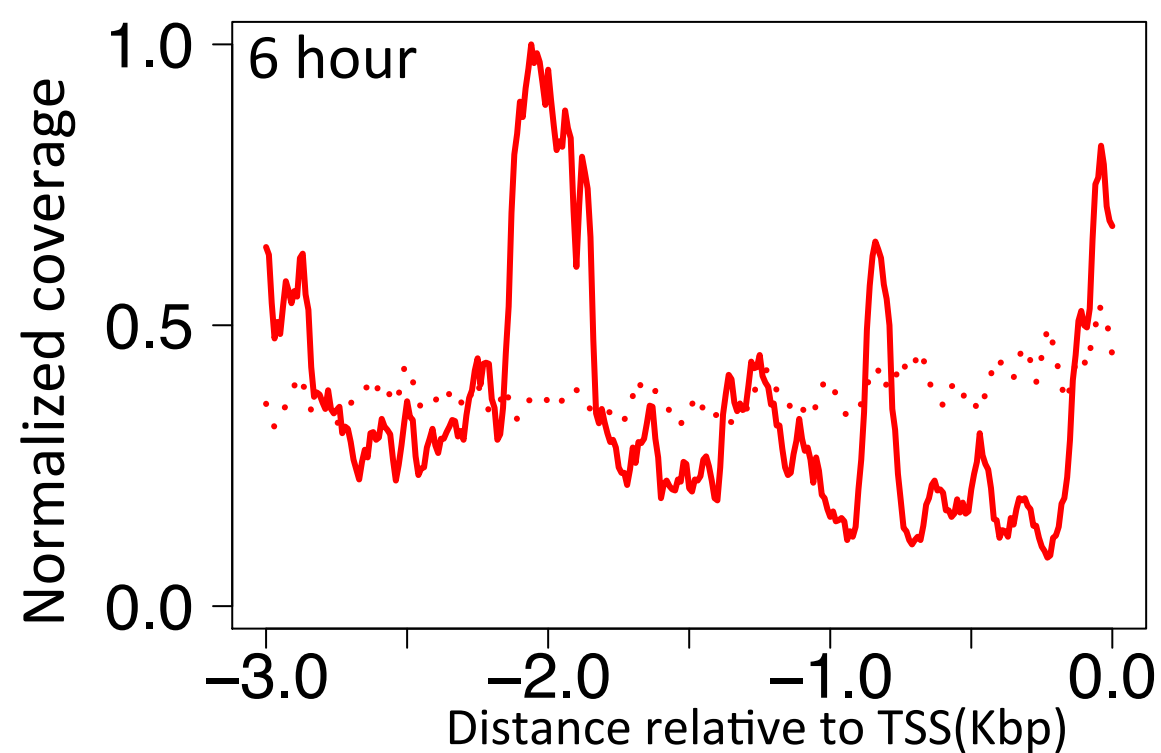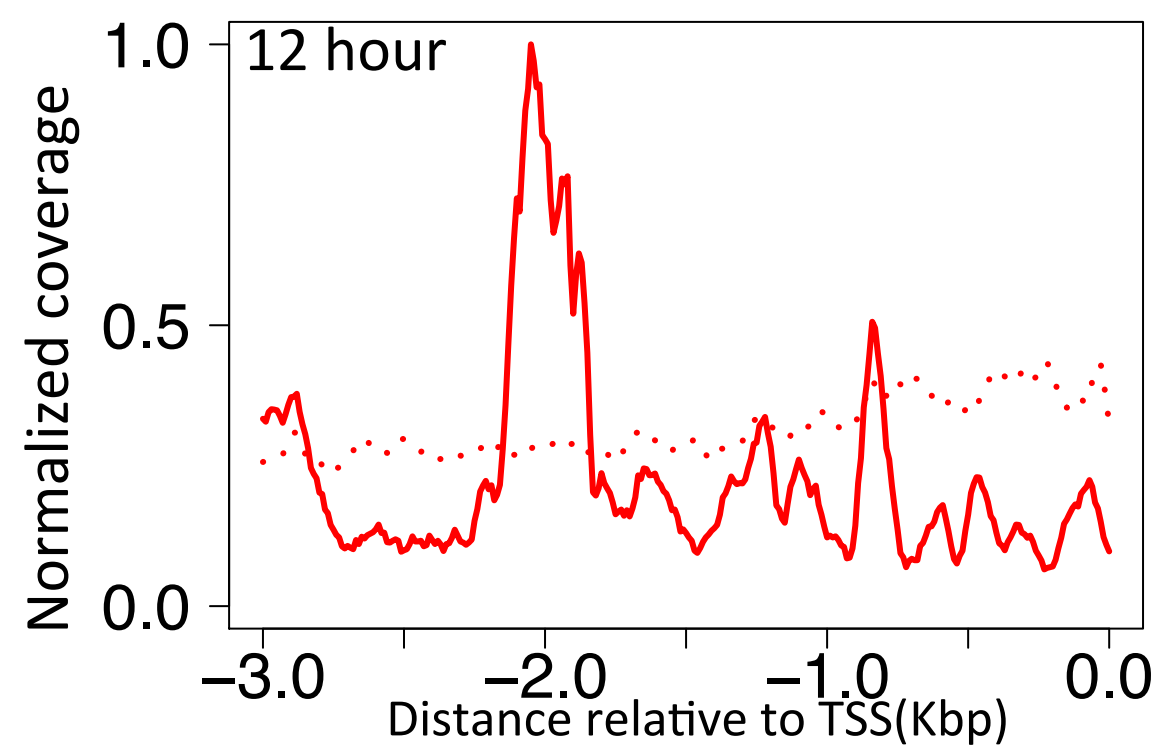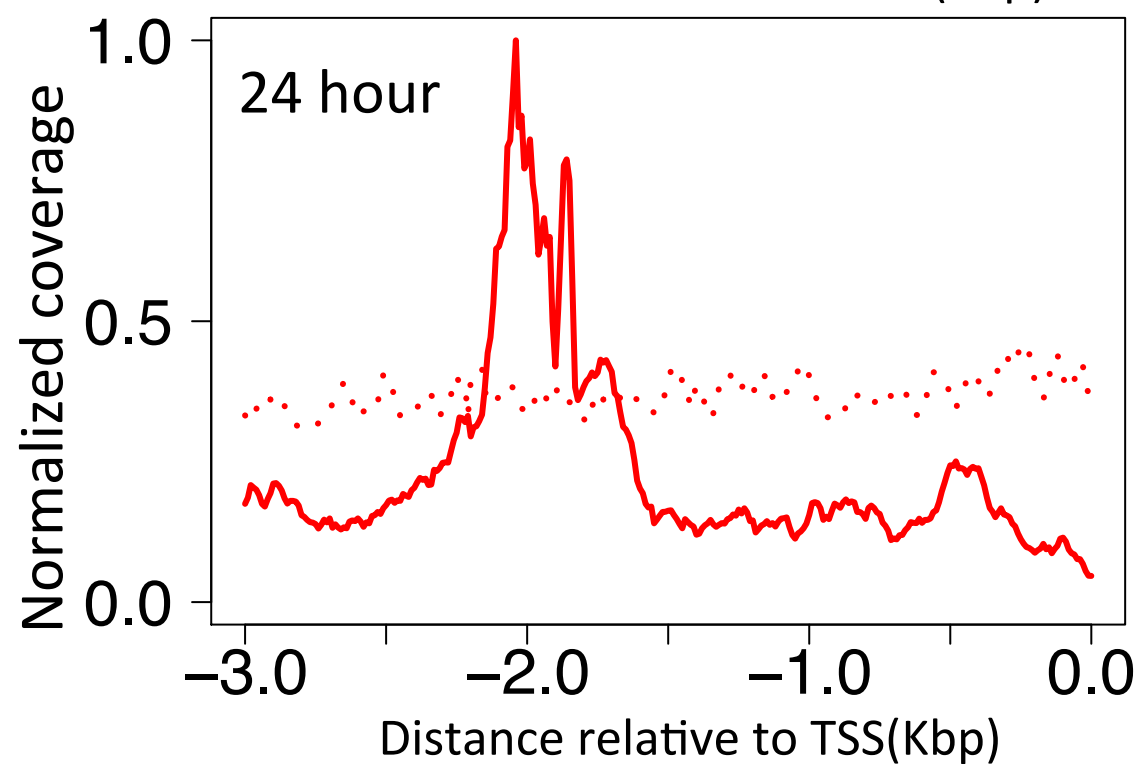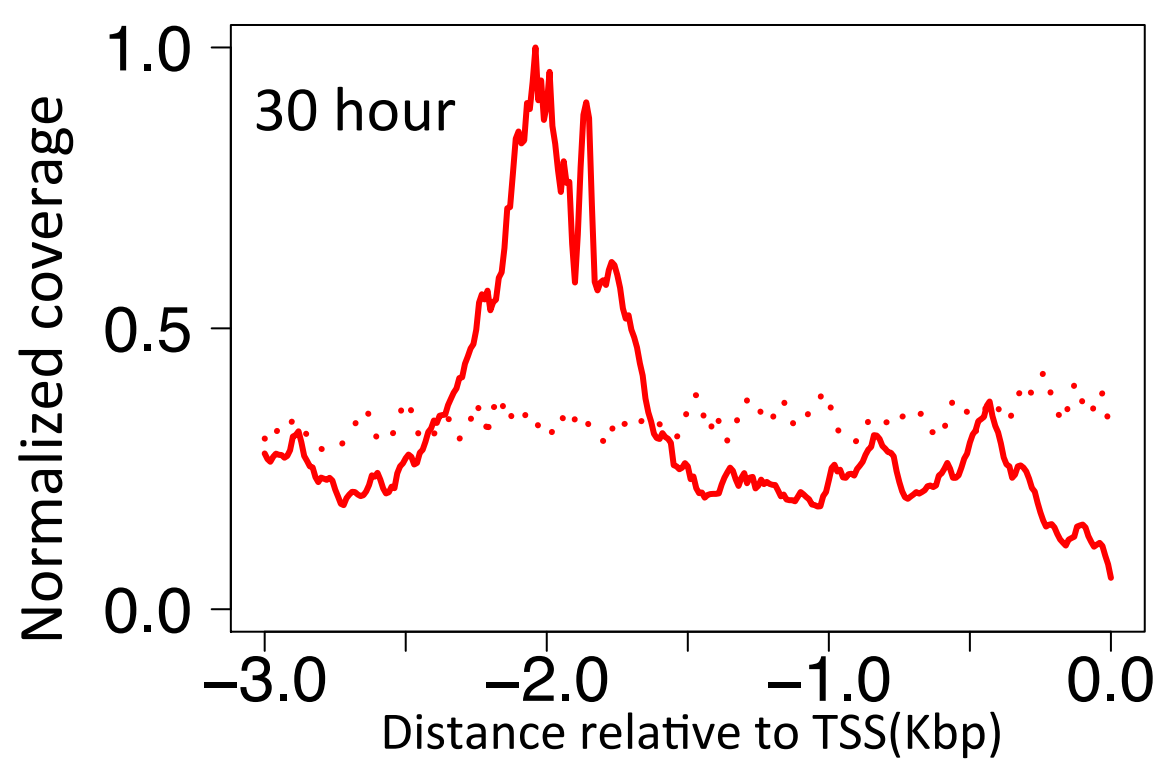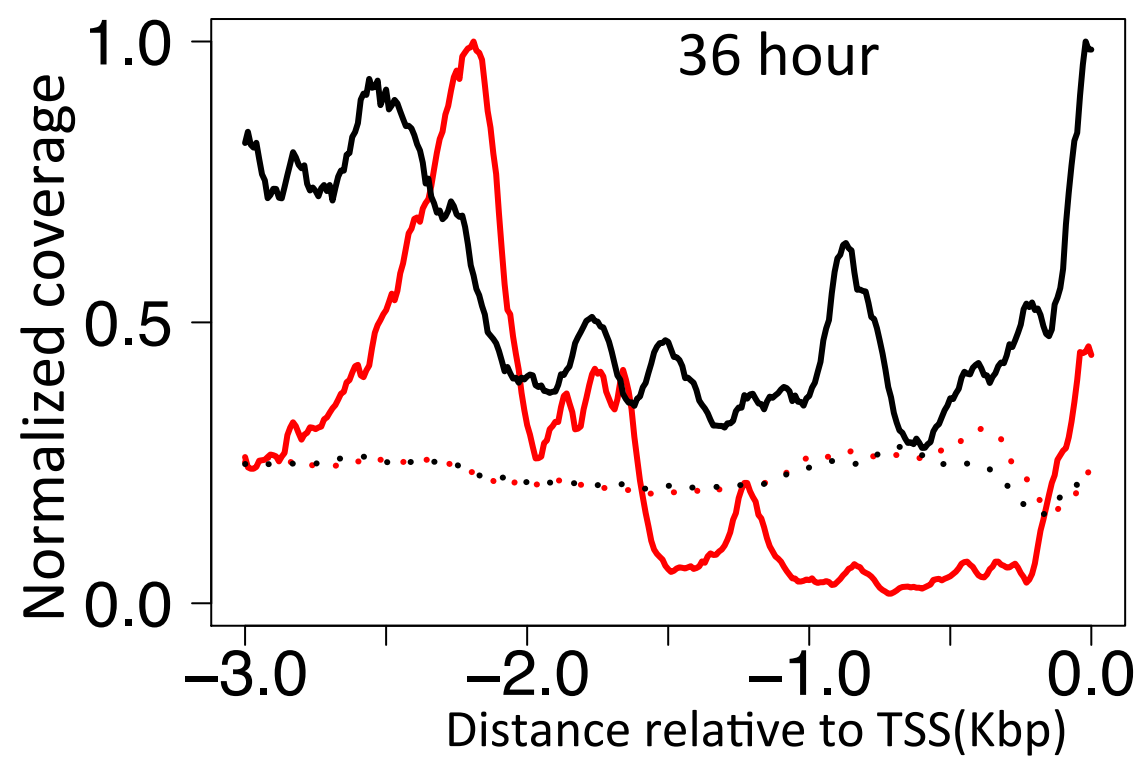

— *Var* FAIRE-Seq      — *Var* MNase-Seq  
· · · Control FAIRE-Seq      · · · Control MNase-Seq

Supplement: Additional file 5: Figure S4. — Average genome-wide sequence read coverage on 5′upstream region for hours 6, 12, 24, 30 and 36 (MNase-seq data [25] is available for hours 0, 18 and 36). (PDF 102 kb) [file 12864_2016_3005_MOESM5_ESM.pdf]

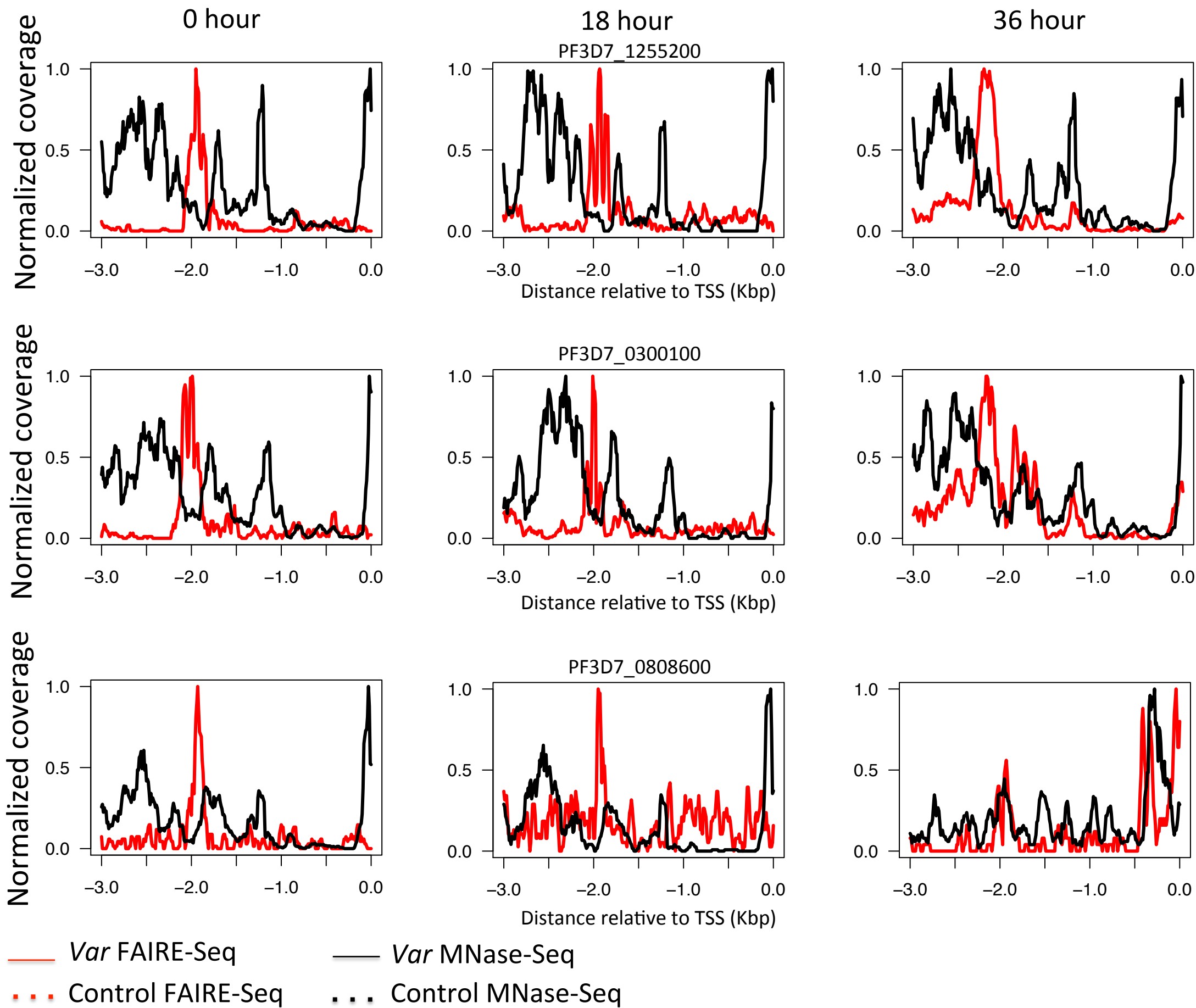

Supplement: Additional file 6: Figure S5. — Boxplot distribution of FAIRE-Seq signal along var 5′ upstream region during stages of ring and trophozoite. (‘****’ represents p-value < 2.2e-16; ‘**’ represents p-value < 0.01; ‘*’ represents p-value < 0.05. P-value was calculated based on Wilcoxon-Rank-Sum test between var gene and the genes with one intron in P. falciparum). (PDF 144 kb) [file 12864_2016_3005_MOESM6_ESM.pdf]

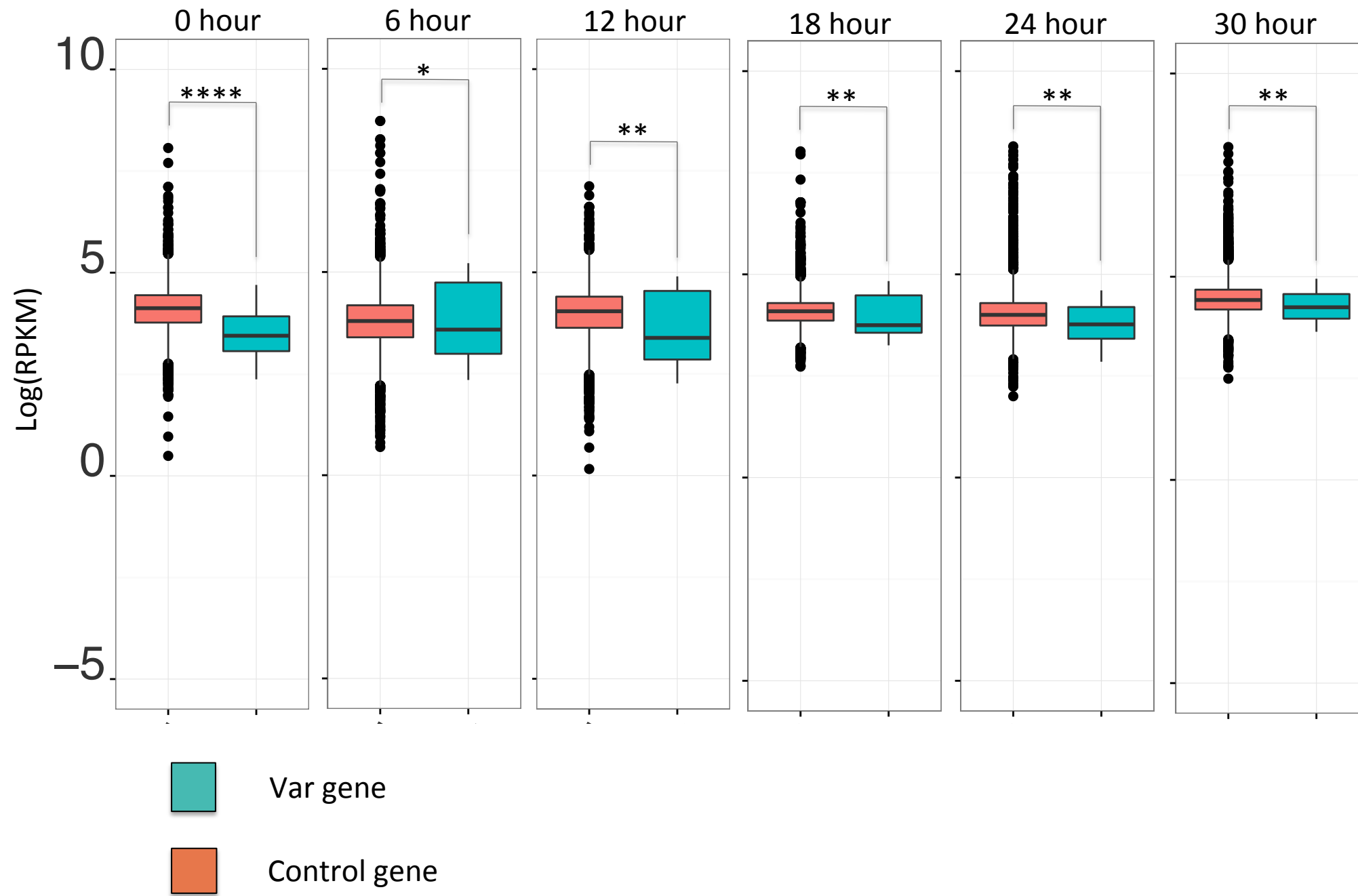

Supplement: Additional file 7: Figure S6. — Average genome-wide sequence read coverage on gene 5′ upstream region for hours 6, 12, 24 and 30 (MNase-seq data [25] is available for hours 36). (PDF 126 kb) [file 12864_2016_3005_MOESM7_ESM.pdf]

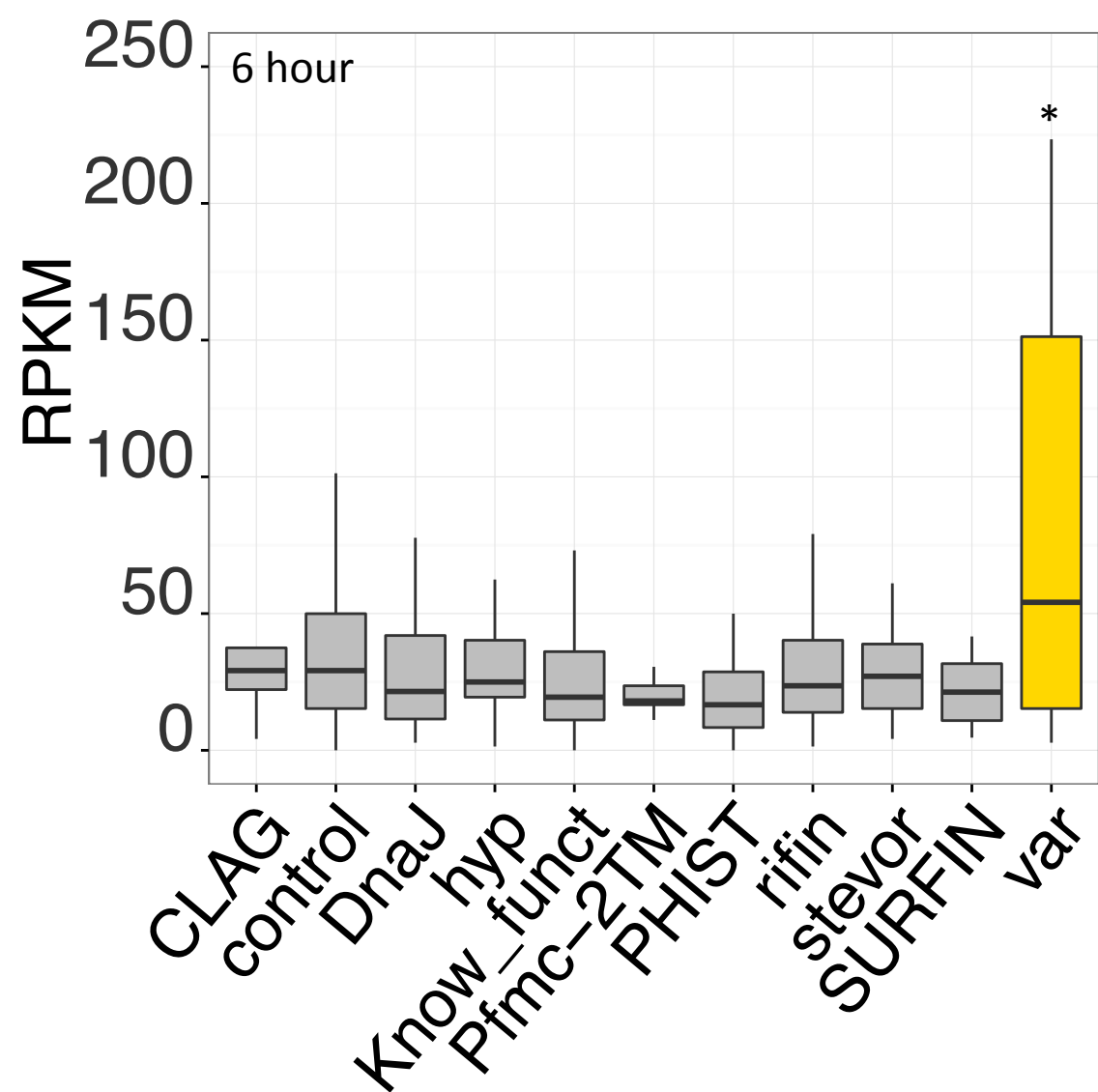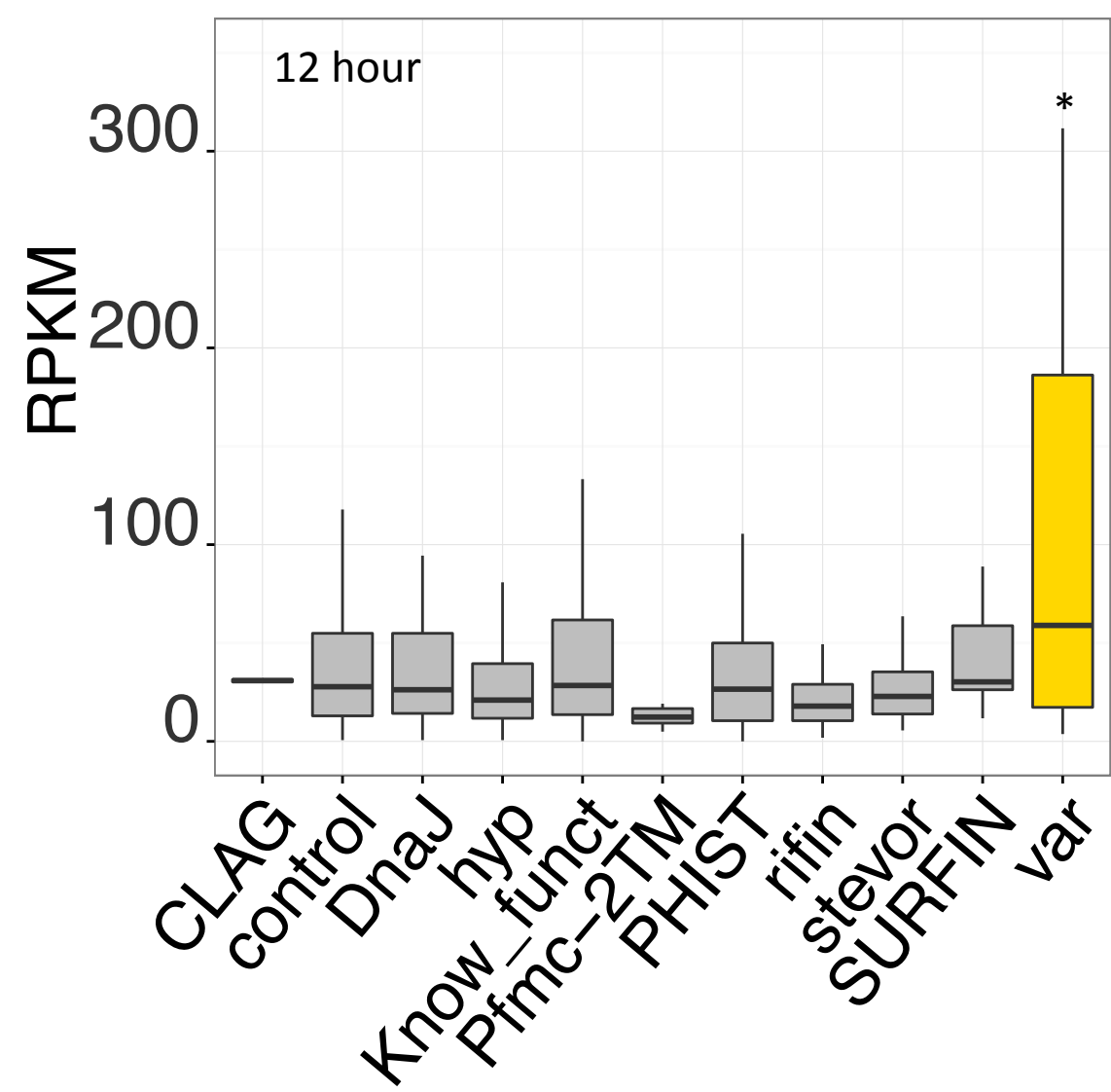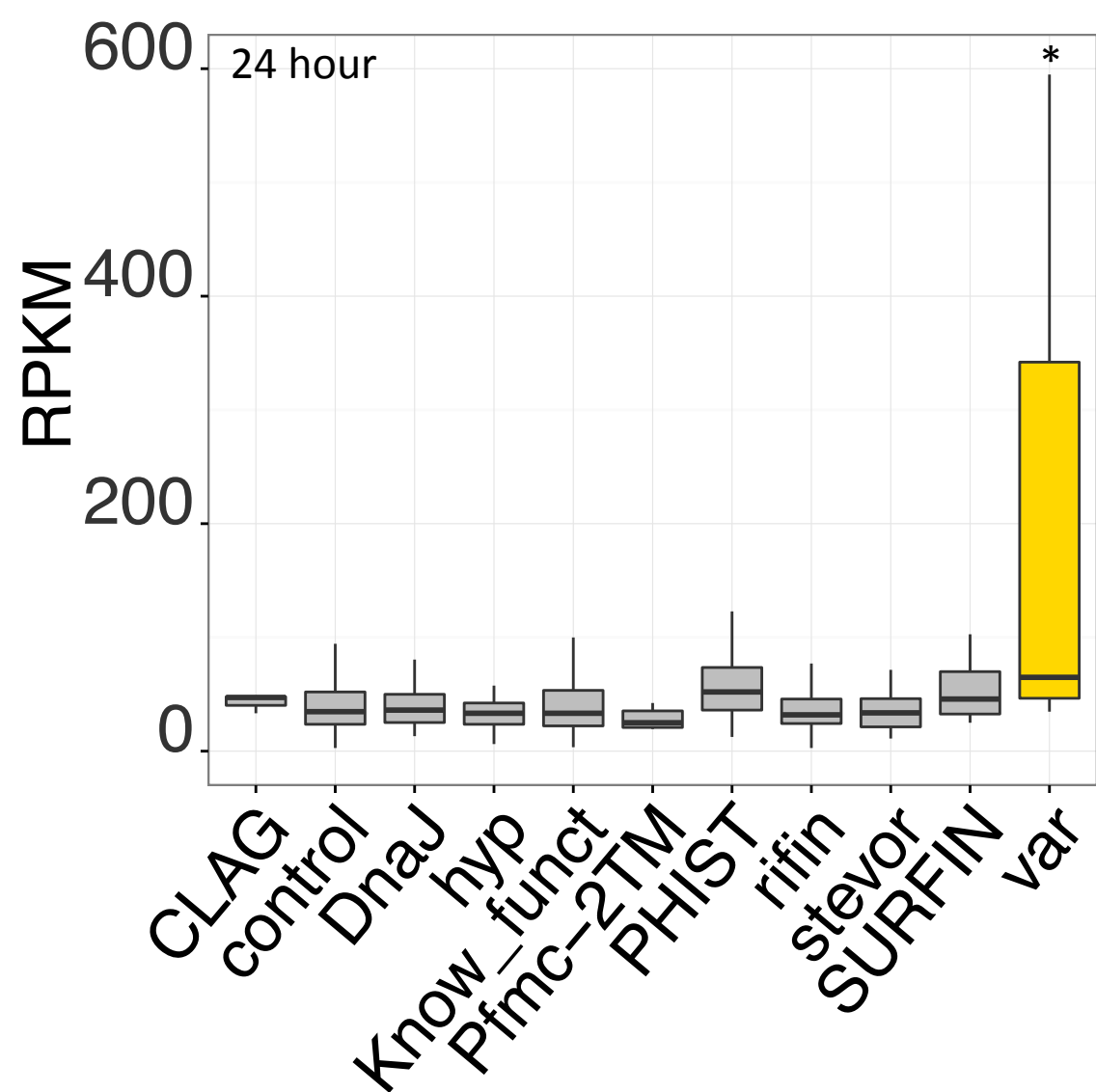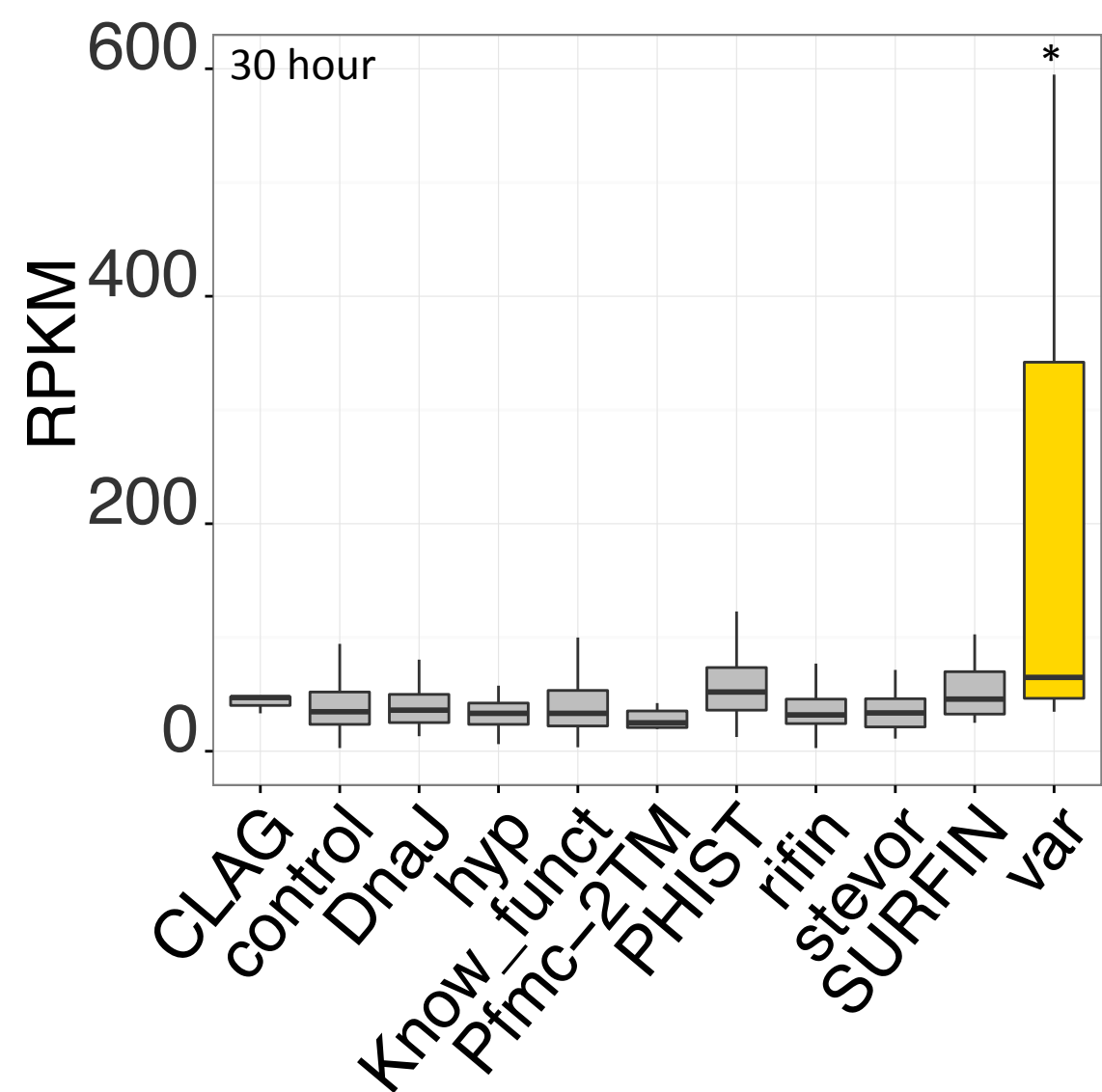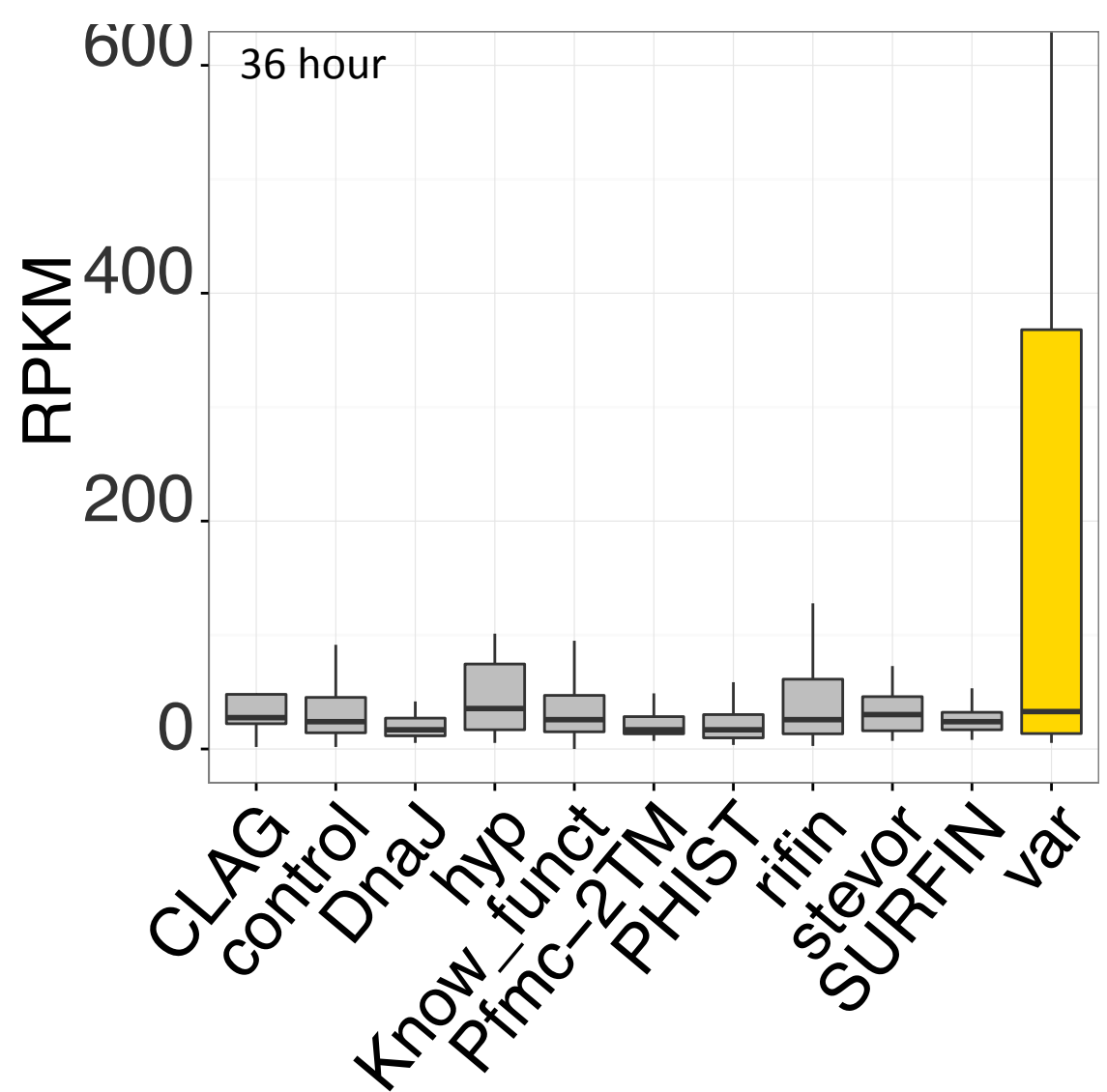

Supplement: Additional file 8: Figure S7. — Boxplot shows FAIRE-Seq signal around 2 kb upstream regions grouped according to gene annotation (‘*’ represents p-value < 0.05 and FDR < 0.05 for all comparisons between different gene groups and var genes). (PDF 97 kb) [file 12864_2016_3005_MOESM8_ESM.pdf]

Bunnik et al. [20] (RNA-Seq)

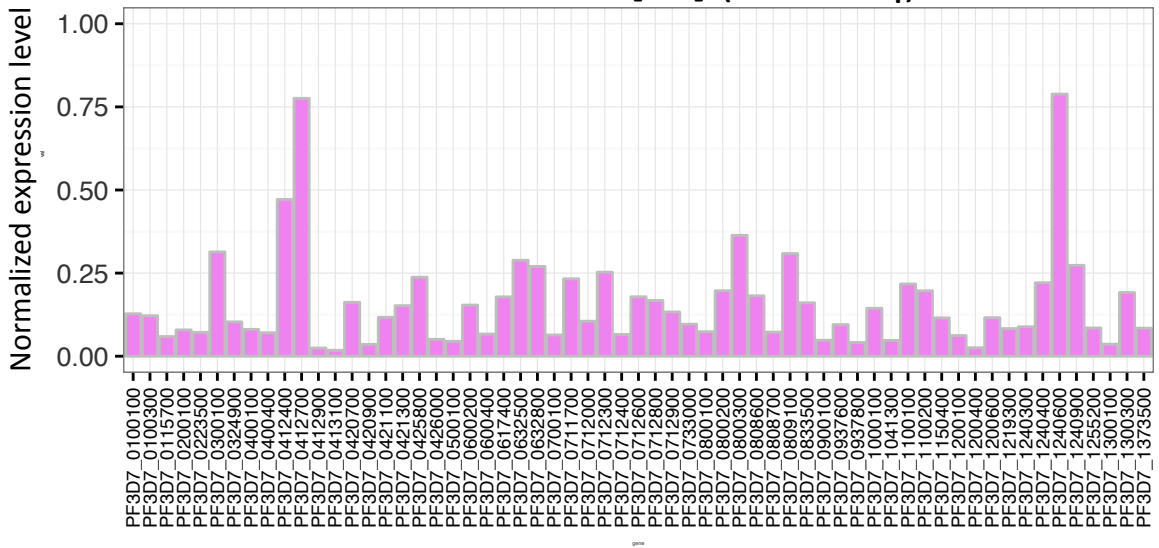

## Otto TD et al.[27] (RNA-Seq)

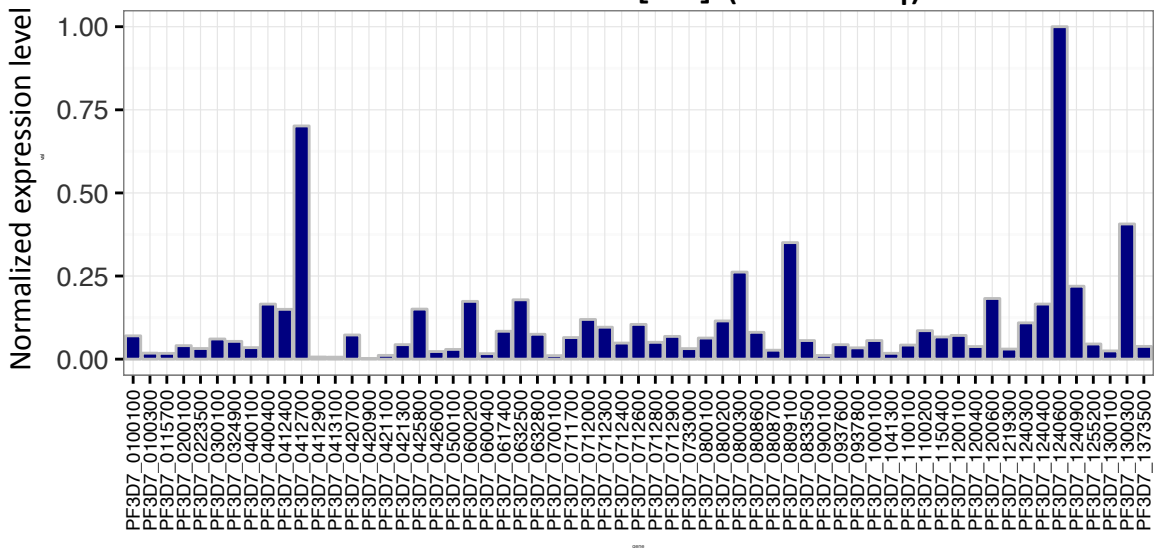

Lopez-Barragan et al[28]. (cDNA)

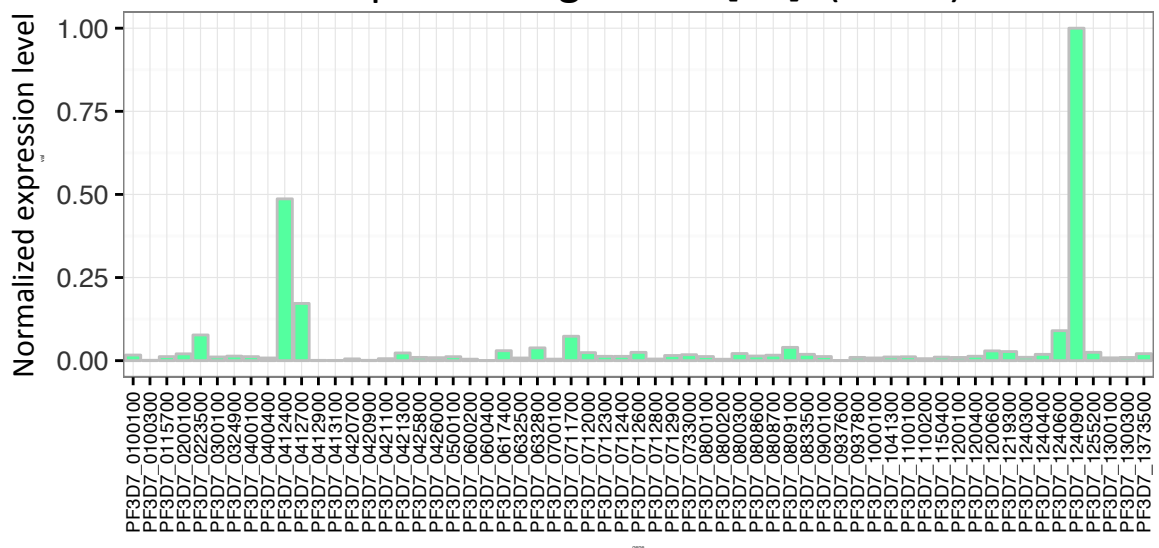

Supplement: Additional file 10: Figure S8. — Bar plots show expression level of var genes during ring stage from three independent data sets. Expression level is expressed as the fraction of the highest expression value among all var genes. (PDF 55 kb) [file 12864_2016_3005_MOESM10_ESM.pdf]

**a**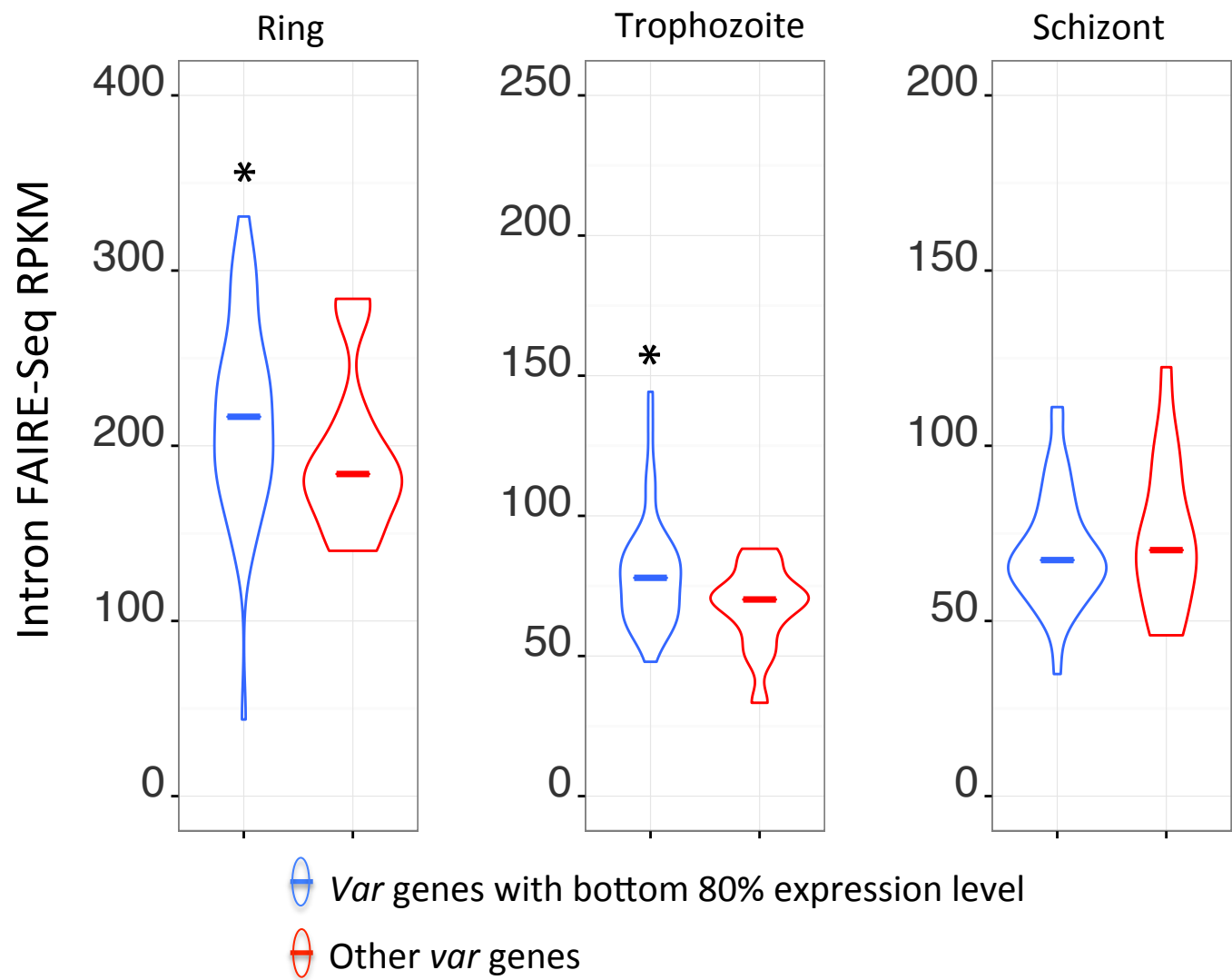**b**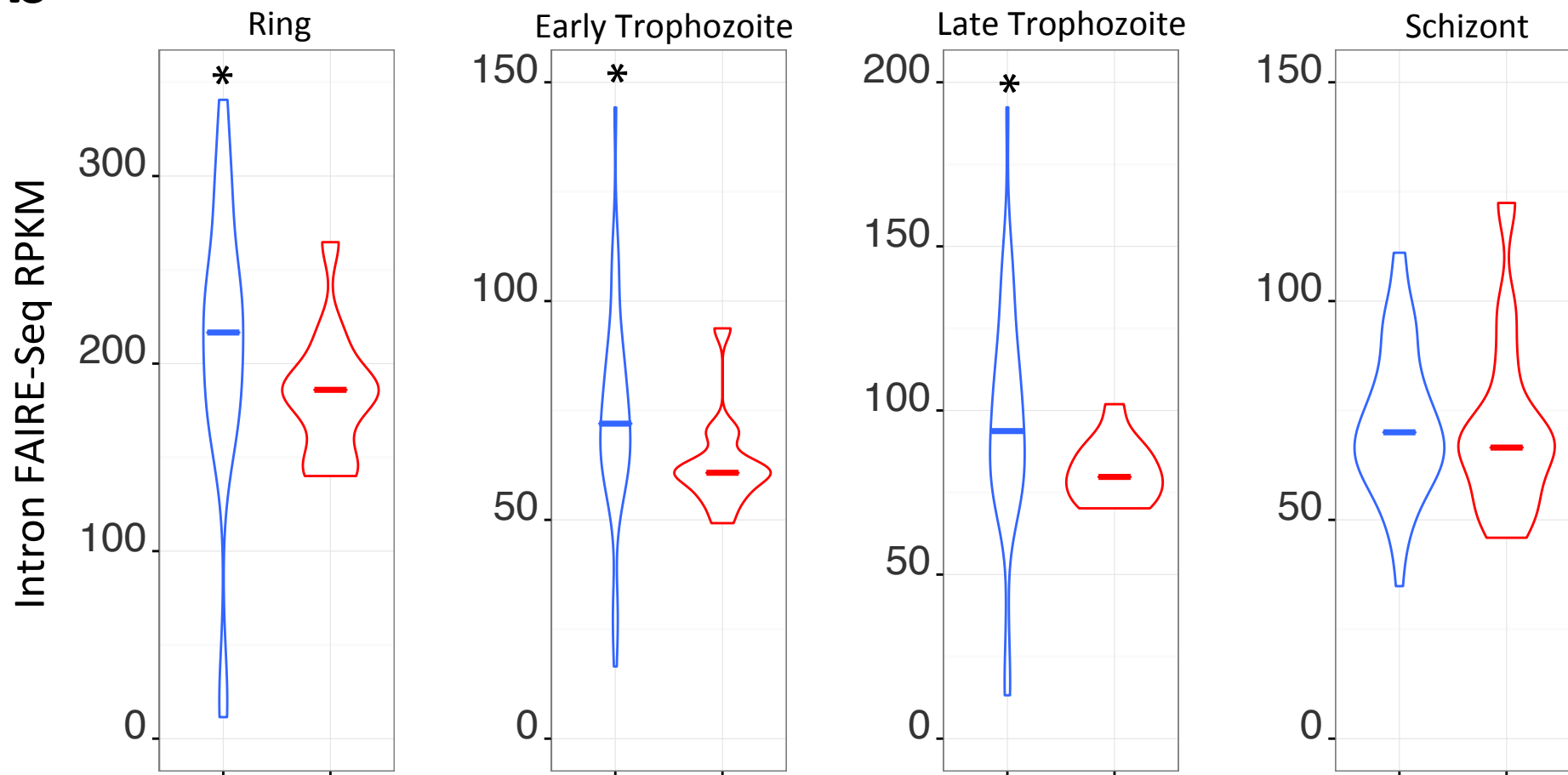

Supplement: Additional file 11: Figure S9. — Boxplot shows the FAIRE-Seq signals var intron regions that are classified based on gene expression data a. Bunnik et al. [20] (RNA-Seq). b. Lopez-Barragan et al. [28] (cDNA) (PDF 142 kb) [file 12864_2016_3005_MOESM11_ESM.pdf]

**a**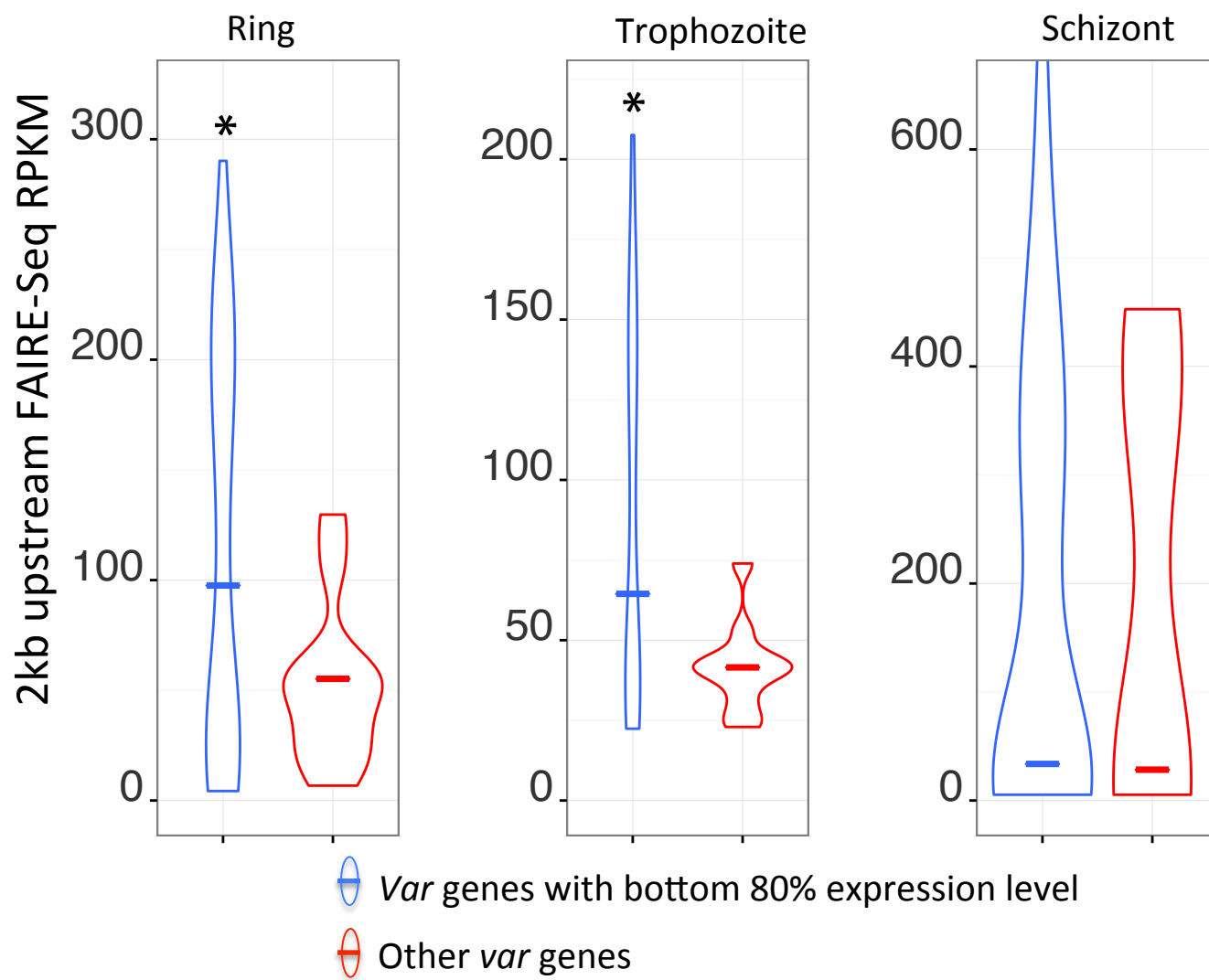**b**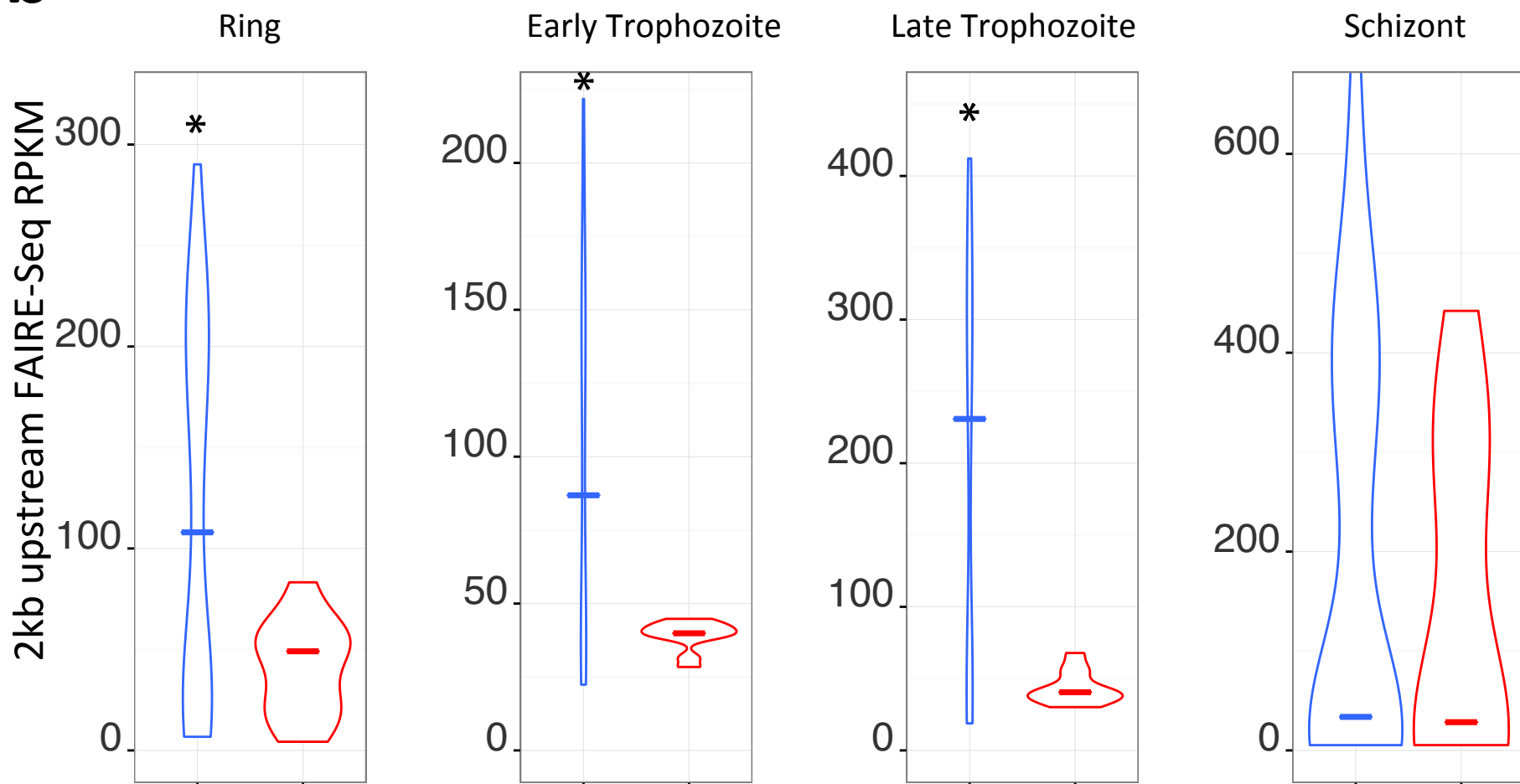

Supplement: Additional file 12: Figure S10. — Boxplot shows the FAIRE-Seq signals var 2 kb upstream regions that are classified based on gene expression data a. Bunnik et al. [20] (RNA-Seq). b. Lopez-Barragan et al. [28] (cDNA) (PDF 143 kb) [file 12864_2016_3005_MOESM12_ESM.pdf]

FAIRE-Seq signal correlation  
between 5'upstream region and intron

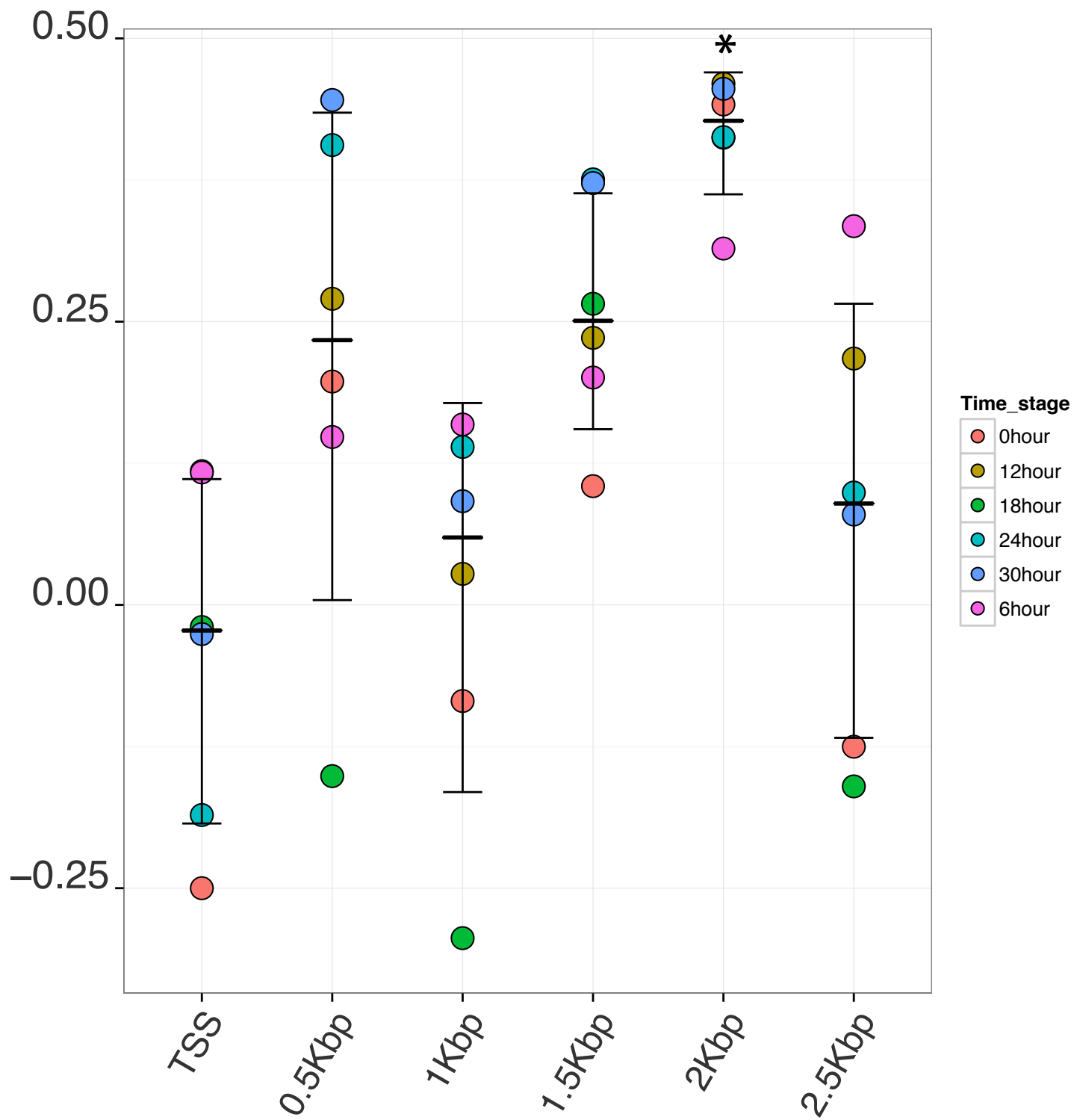

Supplement: Additional file 15: Figure S11. — The bee swarm plot shows Pearson correlation between FAIRE-Seq signal and different var intron and 5′ upstream region signals. (PDF 41 kb) [file 12864_2016_3005_MOESM15_ESM.pdf]

**a**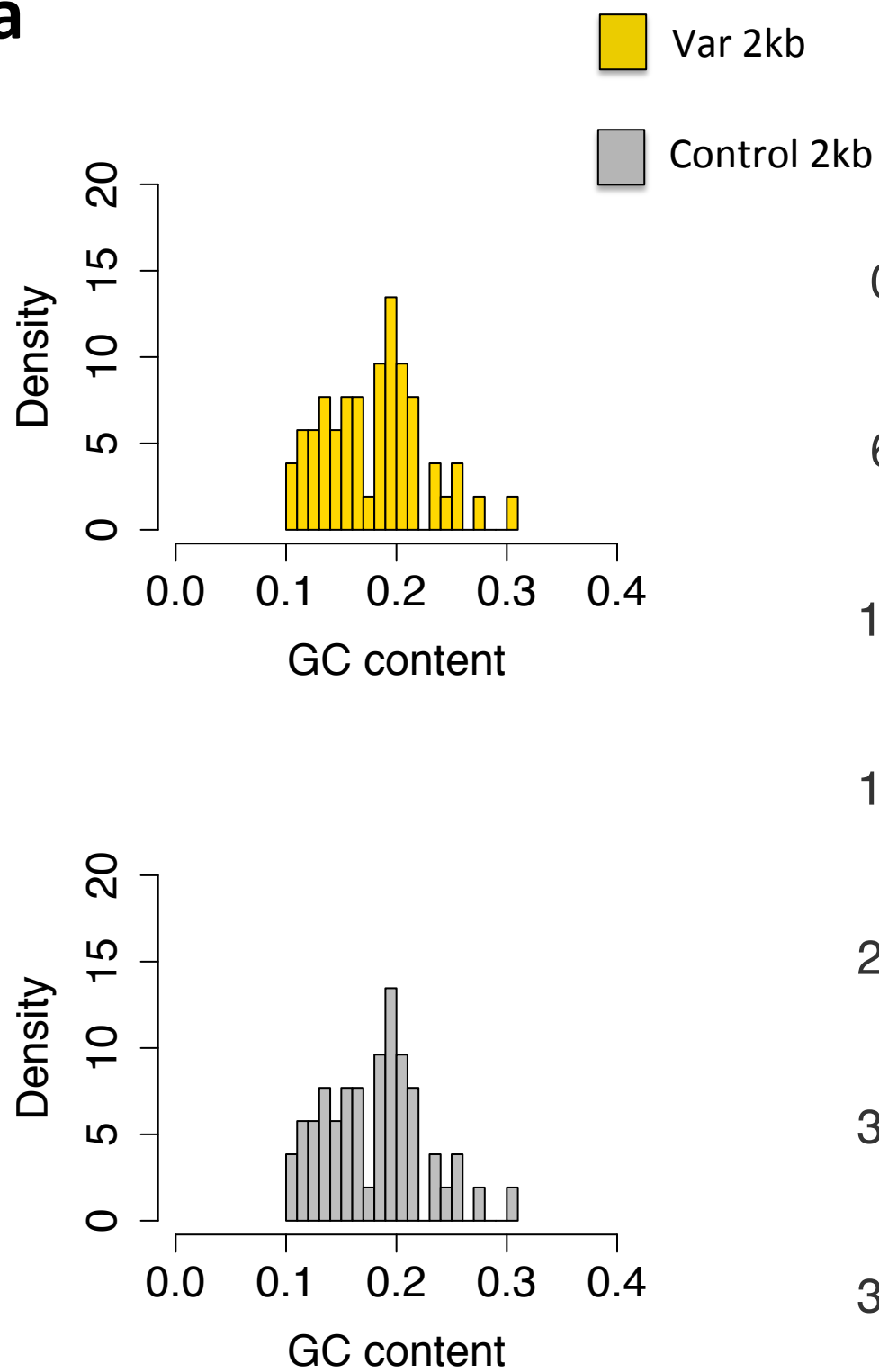**b**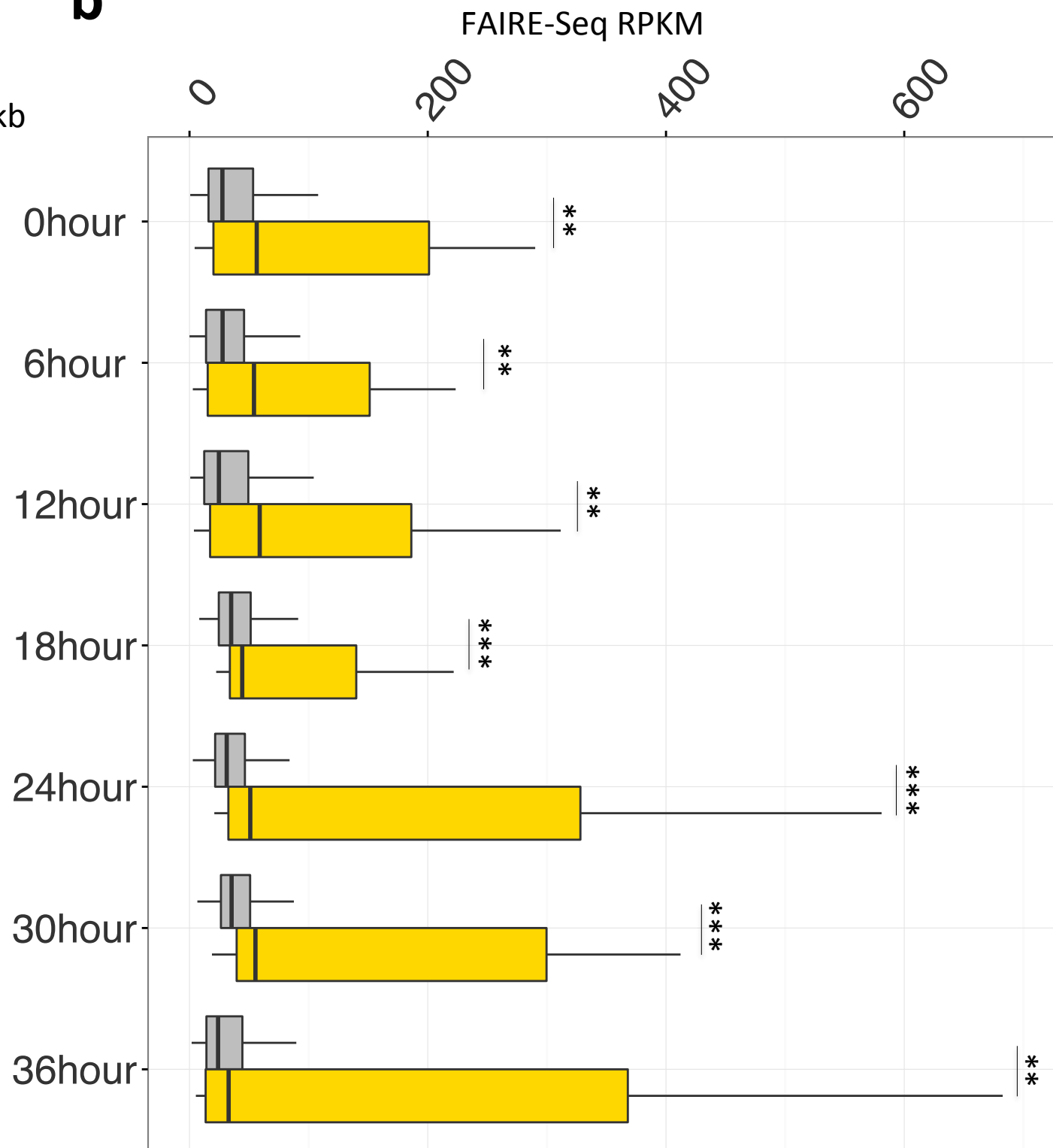

Supplement: Additional file 17: Figure S13. — FAIRE-Seq signal distribution on 2 kb upstream region comparison between var and control genes. Control genes were extracted from the genes in P. falciparum with only one intron and based on the GC content distribution of var 2 kb upstream region. a. The GC content distribution on 2 kb upstream region of var genes and control genes. b. Boxplot distribution shows var 2 kb upstream regions exhibit significantly higher FAIRE-Seq signal compared with control 2 kb upstream region (‘**’ represents p-value < 0.01; ‘***’ represents p-value < 1e-10; P-value was calculated based on Wilcoxon-Rank-Sum test). (PDF 58 kb) [file 12864_2016_3005_MOESM17_ESM.pdf]

a

- 1.1e-192
- 50 sites

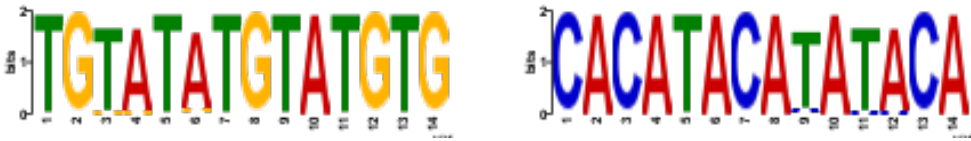

b

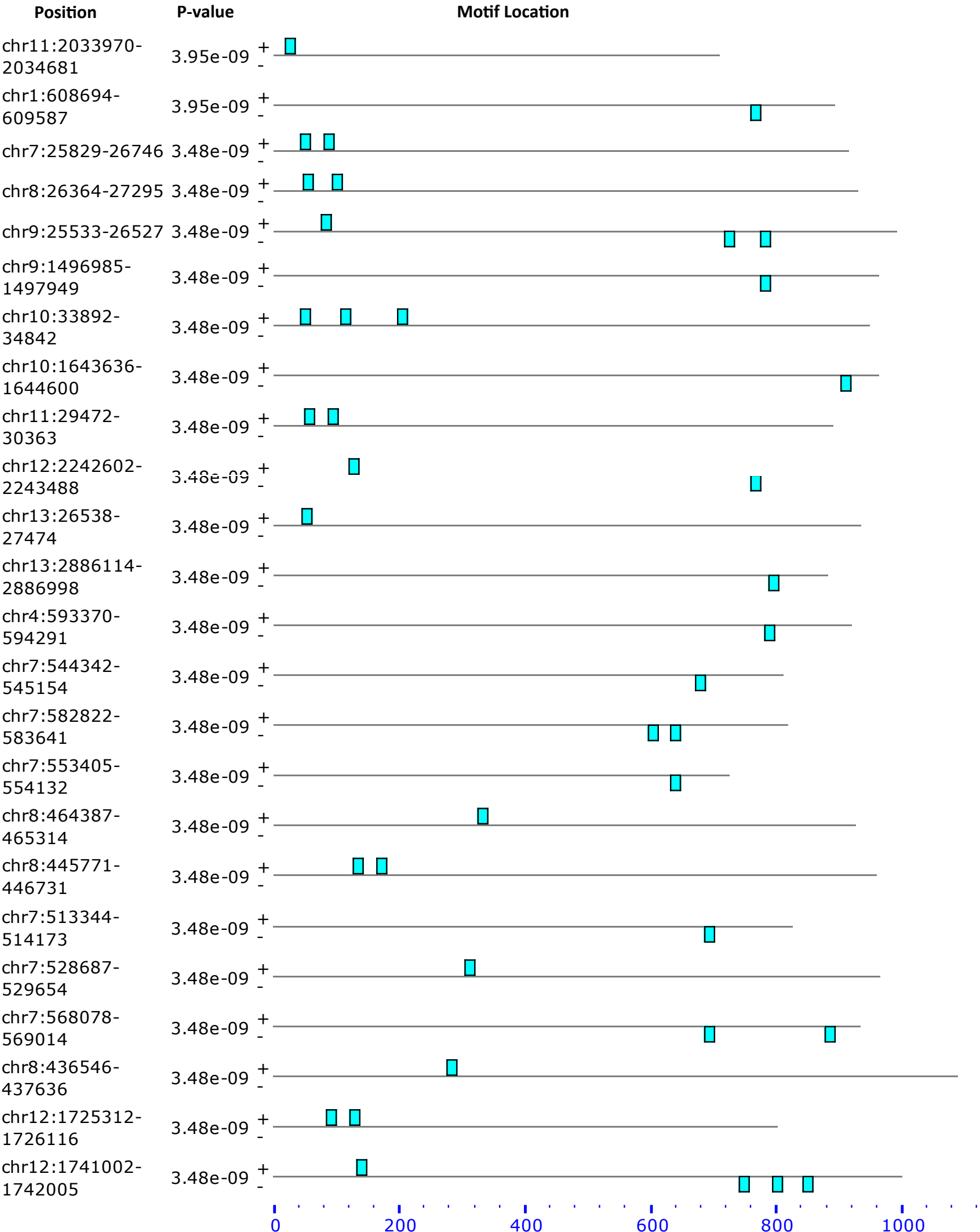

Supplement: Additional file 20: Figure S14. — a. The sequence motif on var introns is discovered by MEME algorithm. b. Significant sequence motif locations in var intron regions. (PDF 277 kb) [file 12864_2016_3005_MOESM20_ESM.pdf]

a

- 8.1e-282
- 50 sites

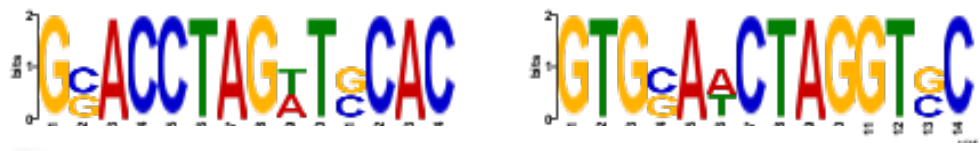

b

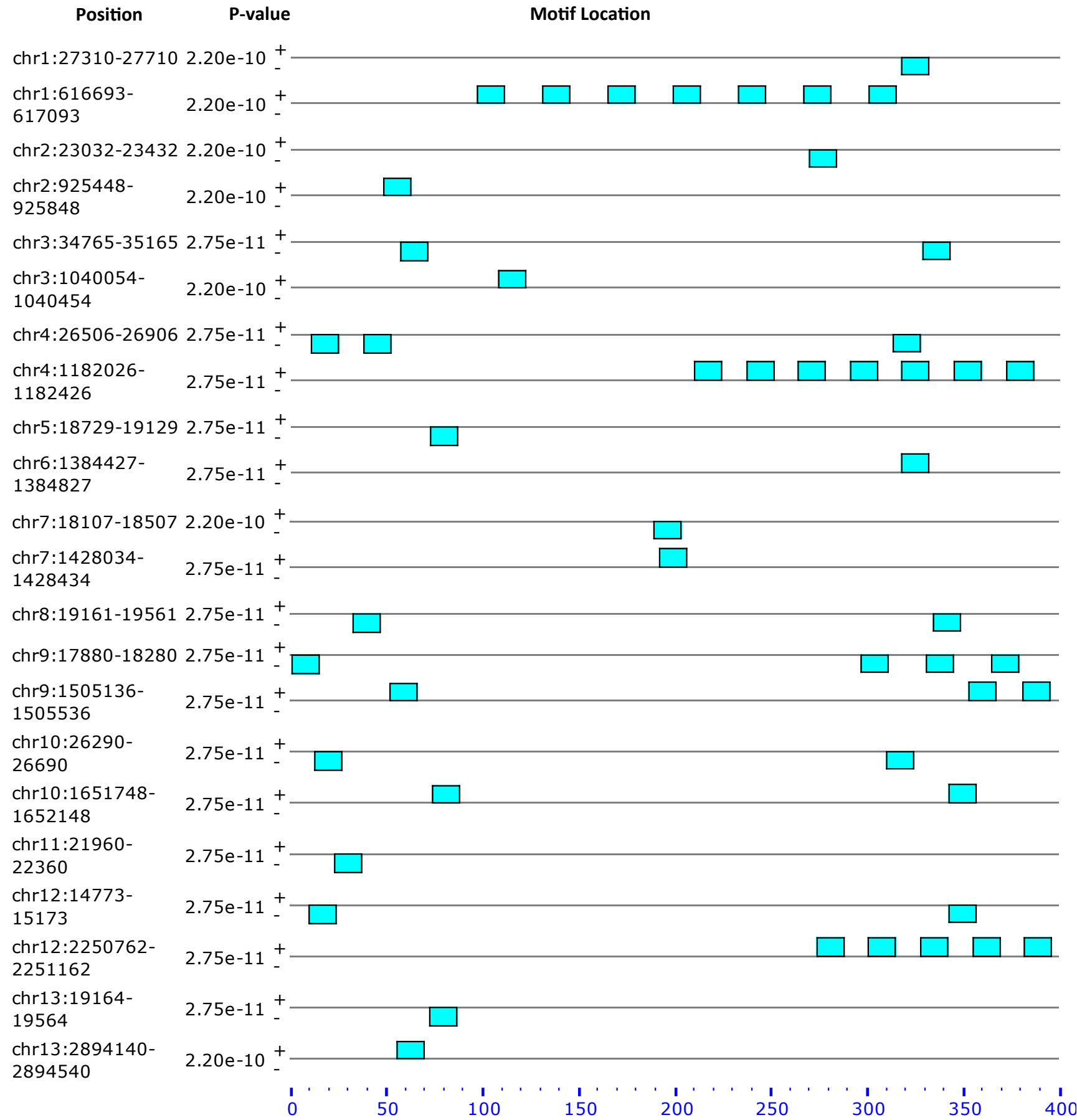

Supplement: Additional file 21: Figure S15. — a. The sequence motif on var 2 kb upstream regions is discovered by MEME algorithm. b. Significant sequence motif locations in 2 kb upstream regions [−200, +200]. (PDF 227 kb) [file 12864_2016_3005_MOESM21_ESM.pdf]
