# Supplementary material for: Punctuated chromatin states regulate Plasmodium falciparum antigenic variation at the intron and 2 kb upstream regions
Source: BMC Genomics. 2016 Aug 18;17:652. doi: 10.1186/s12864-016-3005-7 (PMC4990864; doi:10.1186/s12864-016-3005-7)
Supplement: Additional file 16: Figure S12. — FAIRE-Seq signal distribution comparison between var introns and control introns. Control introns were extracted from the genes in P. falciparum with only one intron and based on the GC content distribution of var introns. a. The GC content distribution of var introns and control introns. b. Boxplot distribution shows var introns exhibit significantly higher FAIRE-Seq signal compared with control introns (‘****’ represents p-value < 2.2e-16; P-value was calculated based on Wilcoxon-Rank-Sum test). (PDF 57 kb) [file 12864_2016_3005_MOESM16_ESM.pdf]

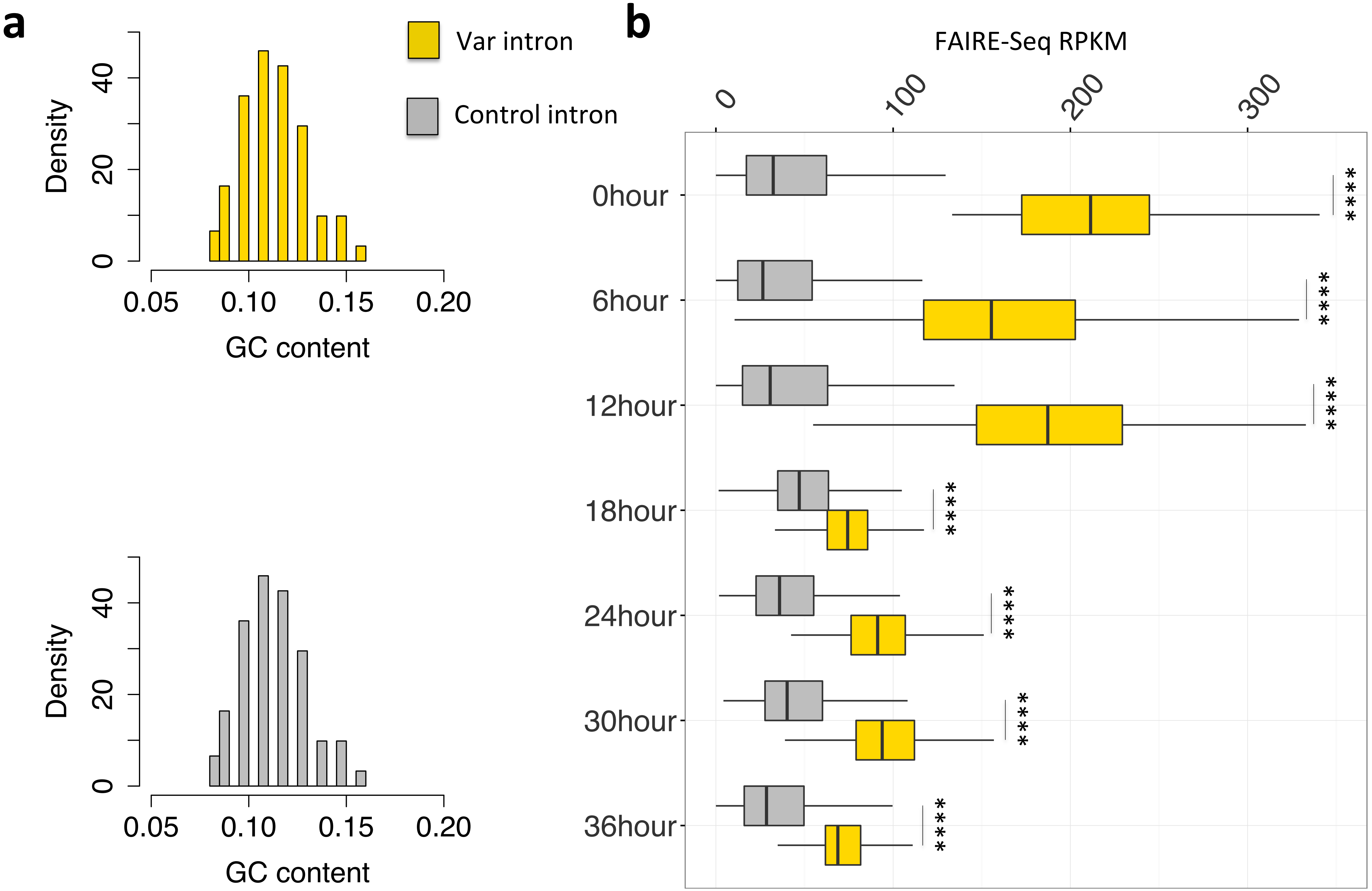
a
Var intron
Control intron
Density
0
20
40
0.05
0.10
0.15
0.20
GC content
b
FAIRE-Seq RPKM
0
100
200
300
0hour
6hour
12hour
18hour
24hour
30hour
36hour
****
